# Spin-Crossing in the (*Z*)-Selective Alkyne Semihydrogenation Mechanism Catalyzed by Mo_3_S_4_ Clusters: A Density Functional Theory Exploration

**DOI:** 10.1021/acs.inorgchem.3c03057

**Published:** 2024-01-04

**Authors:** María Gutiérrez-Blanco, Andrés G. Algarra, Eva Guillamón, M. Jesús Fernández-Trujillo, Mónica Oliva, Manuel G. Basallote, Rosa Llusar, Vicent S. Safont

**Affiliations:** † Departament de Química Física i Analítica, Universitat Jaume I, Av. Sos Baynat s/n, Castelló 12071, Spain; ‡ Departamento de Ciencia de los Materiales e Ingeniería Metalúrgica y Química Inorgánica, Instituto de Biomoléculas (INBIO), Facultad de Ciencias, Universidad de Cádiz, Apartado 40, Puerto Real, Cádiz 11510, Spain

## Abstract

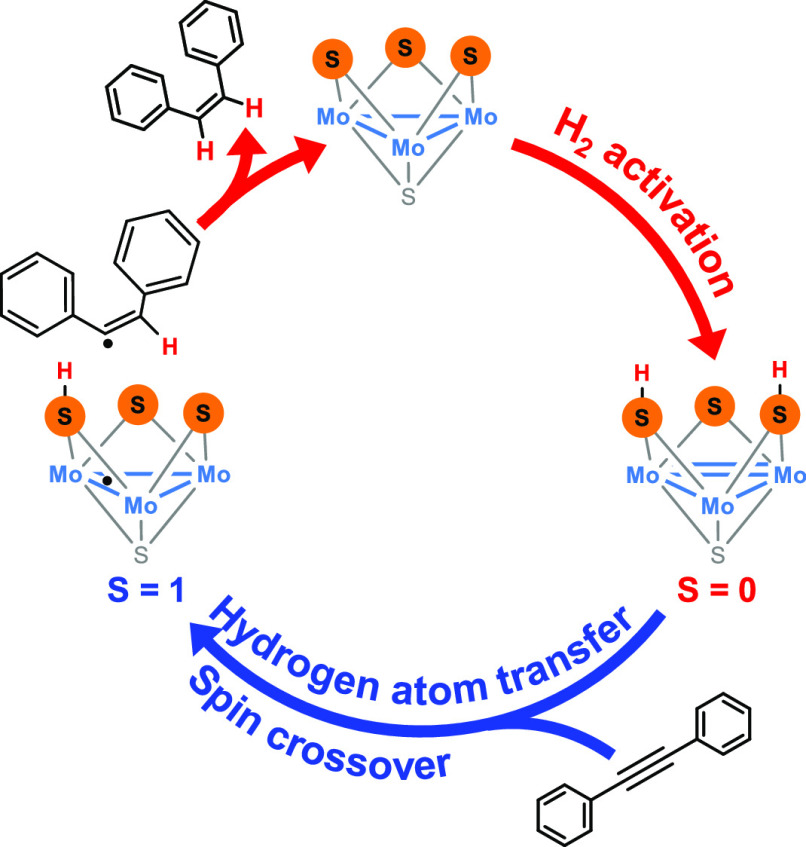

Semihydrogenation of internal alkynes catalyzed by the air-stable imidazolyl amino [Mo_3_S_4_Cl_3_(ImNH_2_)_3_]^+^ cluster selectively affords the (*Z*)-alkene under soft conditions in excellent yields. Experimental results suggest a sulfur-based mechanism with the formation of a dithiolene adduct through interaction of the alkyne with the bridging sulfur atoms. However, computational studies indicate that this mechanism is unable to explain the experimental outcome: mild reaction conditions, excellent selectivity toward the (*Z*)-isomer, and complete deuteration of the vinylic positions in the presence of CD_3_OD and CH_3_OD. An alternative mechanism that explains the experimental results is proposed. The reaction begins with the hydrogenation of two of the Mo_3_(μ_3_-S)(μ-S)_3_ bridging sulfurs to yield a bis(hydrosulfide) intermediate that performs two sequential hydrogen atom transfers (HAT) from the S–H groups to the alkyne. The first HAT occurs with a spin change from singlet to triplet. After the second HAT, the singlet state is recovered. Although the dithiolene adduct is more stable than the hydrosulfide species, the large energy required for the subsequent H_2_ addition makes the system evolve via the second alternative pathway to selectively render the (*Z*)-alkene with a lower overall activation barrier.

## Abstract 2

Selectivity matters: An in-depth theoretical study on the semihydrogenation of alkynes to yield the (*Z*)-isomer catalyzed by Mo_3_S_4_ clusters is presented. Computational analysis and experimental results suggest a novel mechanism in which H_2_ addition to two of the bridging sulfides is followed by a hydrogen atom transfer (HAT), which triggers a spin crossover process. This new mechanism rationalizes the experimental results relative to mild reaction conditions, selectivity, and complete deuteration in the presence of *d*_4_-methanol.

## Introduction

The stereoselective catalytic semihydrogenation of internal alkynes into (*E*)- or (*Z*)-alkene isomers is among the most relevant processes in synthetic organic chemistry.^1^ To date, a plethora of heterogeneous and homogeneous metal-based catalysts for internal alkyne semihydrogenation have been reported.^2−5^ In spite of the many advances in the field, there are still some unsolved problems such as overreduction and isomerization.^6,7^ For that reason, most of the current efforts are devoted to rendering selective catalysts working under mild conditions, with these ideally being also inexpensive, atom-economic, and environmentally benign. Thus, the interest to replace noble metals by nonprecious ones together with using hydrogen as reducing agent is notably increasing. Heterogeneous catalysts are usually preferred by industry, but due to the inherent difficulties in obtaining mechanistic information from solid-state catalysts, the use of molecular models capable of mimicking their behavior has become a widespread approach. Homogeneous catalysts also offer a more rational tuning of the catalyst through metal election and ligand design, enabling high stereoselectivity under mild conditions and broad functional group tolerance. In this context, a mechanistic understanding is essential for the development of better catalysts. There are many examples in the literature supporting this statement.^8−10^

Noble metal coordination complexes have been extensively studied and excellent selectivities toward the (*Z*)-isomer were obtained in alkyne hydrogenation and transfer hydrogenation processes.^11,12^ However, the number of base-metal catalysts for the hydrogenation of alkynes into (*Z*)-alkenes using dihydrogen is still very limited. Some recent representative examples include pincer complexes of Mg, Mn, Fe, Co, and Mo containing PN^H^P, PN^H^S, or PNP ligands.^13−17^ Interestingly, mechanistic investigations revealed unique reaction pathways for those pincer complexes containing M-N bonds in which participation of the ligand in the mechanism was crucial. In general terms, their reaction mechanisms can be classified according to the interaction of the hydrogen molecule with the substrate as “inner-sphere” when the substrate is activated by metal coordination or as “outer-sphere” when the substrate is activated in the second coordination sphere rather than by direct interaction with the metal.^18^

Despite the variety of known transition metal–sulfur complexes, only a few have been applied to catalytic hydrogenation reactions.^19^ This is somewhat surprising, taking into consideration the key role of metal–sulfur compounds in biologically relevant hydrogenation processes. For instance, the heterolysis of dihydrogen catalyzed by [NiFe] hydrogenase is likely to proceed through a cooperative H–H bond splitting at the Ni–S bond.^20^ In a seminal work by DuBois and co-workers, the catalytic semihydrogenation of alkynes into (*Z*)-alkene using cyclopentadienyl dinuclear Mo_2_(μ-S)_2_(μ-S_2_CH_2_) complexes in the presence of a Bro̷nsted acid cocatalysts was reported.^21^ These dinuclear complexes react with alkynes to afford dithiolene adducts that upon hydrogenation selectively generate the (*Z*)-alkene. Unfortunately, the efficiency of these systems is limited because excess alkyne inhibits the hydrogenation.

Recent work by us in collaboration with Beller’s group has shown that cuboidal Mo_3_(μ_3_-S)(μ-S)_3_ clusters are active catalysts for the hydrogenation of various organic substrates.^22−25^ Compounds containing the Mo_3_(μ_3_-S)(μ-S)_3_ cluster unit have been widely studied and their chemistry has been recently reviewed.^26^ With a handful of exceptions, Mo_3_S_4_ complexes are electron precise with six cluster skeletal electrons (CSE) for the formation of three metal–metal bonds and a formal oxidation state of IV for the metal atoms. Electrochemical studies show the presence of three sequential one-electron reduction processes (Mo_3_^IV^ ↔ Mo_2_^IV^Mo^III^ ↔ Mo^IV^Mo_2_^III^ ↔ Mo_3_^III^) or two successive two- and one-electron reductions (Mo_3_^IV^ ↔ Mo^IV^Mo_2_^III^ ↔ Mo_3_^III^) depending on the nature of the terminal ligands.^26^ The three metal atoms in the six CSE Mo_3_^IV^ clusters define an equilateral triangle. Reduction to the Mo_2_^IV^Mo^III^ seven CSE cluster causes a substantial elongation (ca. 0.038 Å) of one of the Mo–Mo distances, while the other two remain practically unchanged. DFT studies prove that the origin of this distortion and expansion obeys to electronic factors and localize the unpaired electron in one of the Mo atoms.^27^

**Figure 1:**
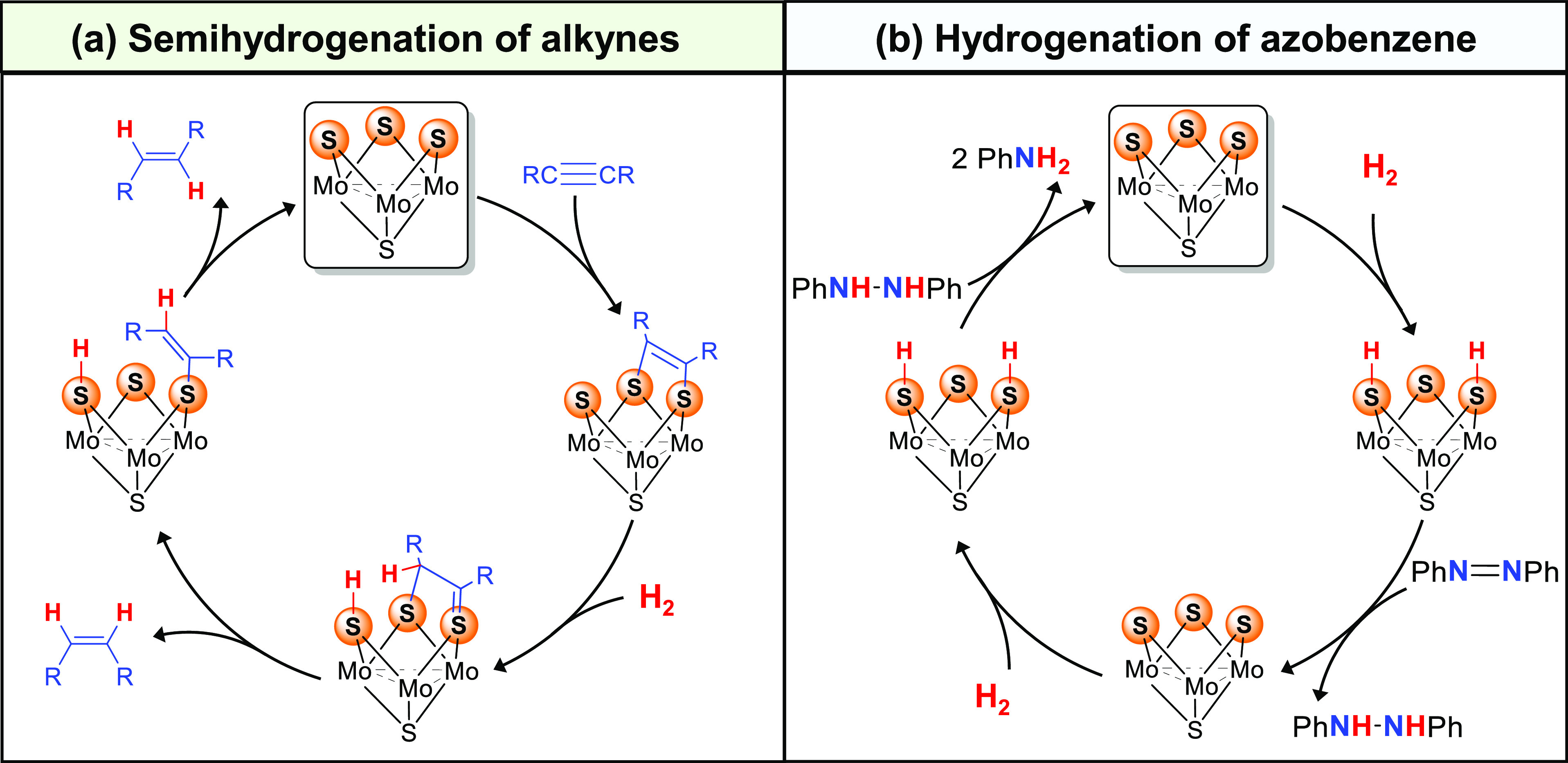
Simplified catalytic cycles for alkyne semihydrogenation (a) and azobenzene hydrogenation (b) in the presence of Mo_3_S_4_ clusters.

Our recent work on alkyne semihydrogenation using Mo_3_S_4_ cluster catalysts has proved that the selectivity toward the (*Z*)-isomer is strongly dependent on the nature of the outer ligand. While the diamino [Mo_3_S_4_Cl_3_(dmen)_3_]^+^ (dmen = Me_2_NCH_2_CH_2_NMe_2_) cluster catalyzes the semihydrogenation of diphenylacetylene (dpa) to produce mixtures of (*Z*)- and (*E*) -alkenes (*Z*/*E ca*. 6/1) under harsh conditions with moderate yields,^28^ the imidazolyl amino [Mo_3_S_4_Cl_3_(ImNH_2_)_3_]^+^ cluster performs the selective transformation toward the (*Z*)-alkene under softer conditions with quantitative yields.^29^ Based on catalytic and stoichiometric experiments, we were able to propose a reaction mechanism for the [Mo_3_S_4_Cl_3_(dmen)_3_]^+^ cluster, depicted in Figure 1a, which starts with the formation of a dithiolene adduct by interaction between the bridging sulfides of the molybdenum cluster complex and the alkyne substrate.

Activation of the alkyne by the trinuclear Mo^IV^_3_(μ_3_-S)(μ-S)_3_ cluster unit occurs without participation of the metal (see Figure 1a). During the course of this [3 + 2] cycloaddition reaction, i.e., alkyne addition to the cluster, the trimetallic unit undergoes a formal two-electron reduction from Mo_3_^IV^ to Mo^IV^Mo_2_^III^. This internal electron transfer causes the shortening of one of the Mo–Mo bonds from 2.759 Å, characteristic of a single bond, to 2.653 Å, typical of a double bond. Therefore, the resulting Mo_3_S_4_ dithiolene cluster contains eight CSE for the formation of two single and one double Mo–Mo bonds.^28^ In the next step, H_2_ activation occurs at the third bridging sulfur and one of the dithiolene carbon atoms, as shown in Figure 1a. This mechanism cannot be categorized as an inner or outer sphere mechanism, and it is better framed within the category of reductive activation as opposed to oxidative and redox neutral activation.^30^ This last classification was recently proposed by Poli based on how the H_2_ molecule is activated and transferred to the catalysts and how they alter (or not) the metal formal oxidation state. Although formally speaking, a reductive activation mechanism entails the transfer of both hydrogens as protons, thus resulting in a two-electron reduction of the metal catalysts, in this case, reduction occurs upon alkyne cycloaddition, and hydrogens are formally transferred as a proton and a hydride. After H_2_ activation, the half-hydrogenated intermediate renders the desired (*Z*)-alkene or isomerizes into an analogue from which (*E*)-alkene is released. The relative energies of these two processes, (*Z*)-hydrogenation vs. (*E*)-isomerization, determine the stereoselectivity of the process.

Cubane-type Mo^IV^_3_(μ_3_-S)(μ-S)_3_ clusters can also activate H_2_ via direct interaction of the hydrogen atoms with two of the bridging sulfurs to form a Mo^IV^Mo_2_^III^(μ_3_-S)(μ–S-H)_2_(μ-S) intermediate containing two hydrosulfide groups. We have postulated this mechanism for the catalyzed hydrogenation of azobenzene by the diamino [Mo_3_S_4_Cl_3_(dmen)_3_]^+^ cluster cation (see Figure 1b) on the basis of kinetic, stoichiometric, and catalytic experiments combined with DFT calculations.^31^ In our proposal, H_2_ delivers two protons to two of the bridging Mo_3_S_4_ sulfur atoms while the trimetallic units gets reduced by two electrons from Mo_3_^IV^ to Mo^IV^Mo_2_^III^ so a dihydrogen reductive activation mechanism, according to Poli’s classifications, operates in this case. Next, the bis(hydrosulfido) intermediate can transfer both hydrogen atoms to azobenzene and to 1,2-diphenylhydrazine to finally afford aniline through two interconnected cycles with similar rate constants. Incidentally, a similar H_2_ activation mechanism has been suggested for the hydrogenation of azo compounds using cyclopentadienyl dinuclear Mo_2_(μ-S)_2_(μ-S_2_CH_2_) complexes, although the intimate mechanism of the hydrogen transfer to the azo substrate remains unclarified.^32^

Motivated by the excellent performance of the imidazolyl amino [Mo_3_S_4_Cl_3_(ImNH_2_)_3_]^+^ cluster catalyst for the (*Z*)-selective semihydrogenation of diphenylacetylene under mild conditions, we decided to undertake a full mechanistic investigation considering the two potential catalytic cycles represented in Figure 1. Despite the relevant differences between both mechanisms, the direct participation of the bridging sulfide ligands in the hydrogenation of unsaturated moieties is a common feature. Nevertheless, while the sulfur sites act as a platform for the unsaturated bond activation during the hydrogenation of alkynes, they are responsible for the H–H bond cleavage during the hydrogenation of azobenzene.^28,31^ To our surprise, the results point to a mechanism involving the initial activation of H_2_ at the μ-S ligands of the cluster to yield an intermediate capable of undergoing two sequential hydrogen atom transfers to the alkyne that entails a spin crossover between singlet and triplet electronic states. This finding reveals the subtle aspects that control the reactivity of the bridging sulfide ligands in these molybdenum sulfide clusters.

## Results and Discussion

### Mechanism via Alkyne Addition to the Cluster

**Figure 2:**
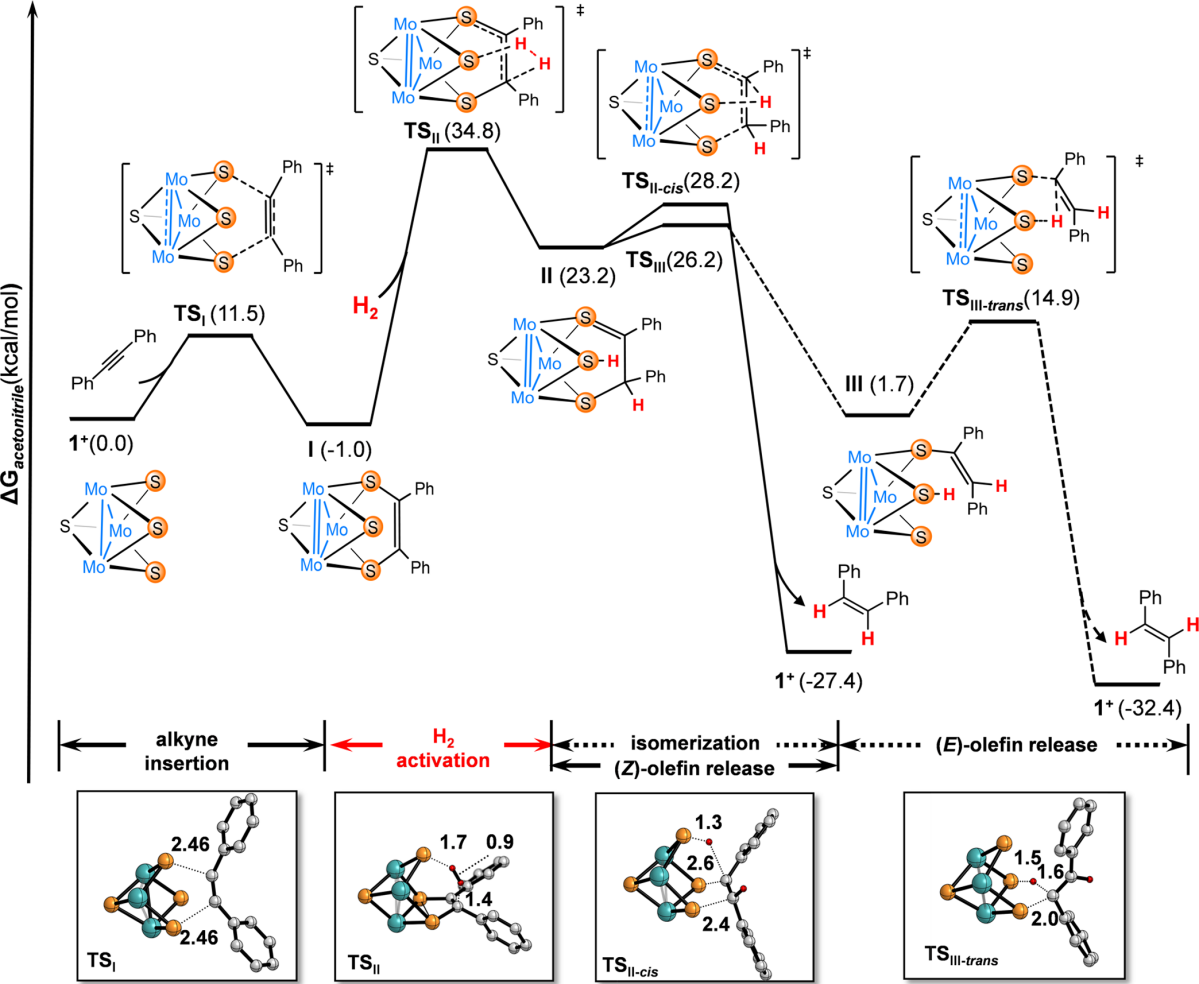
Gibbs energy profile for the semihydrogenation of dpa through a dithiolene-mediated mechanism. Free energy values are given in kcal·mol^–1^, quoted relative to **1**^+^+ dpa + H_2_. Ligands and hydrogen atoms have been omitted for clarity (except vinylic hydrogens). Selected bond distances are given in Å.

As a starting point, we assume that a mechanism analogous to the one previously reported by our group for the semihydrogenation of diphenylacetylene (dpa),^28^ represented in Figure 1a, using a diamino Mo_3_S_4_ cluster catalyst, also operates for the remarkably more active and selective [Mo_3_S_4_Cl_3_(ImNH_2_)_3_]^+^ (**1**^+^) complex.^29^ The computed free energy profile is represented in Figure 2.

The process starts with the [3 + 2] cycloaddition reaction between the two sp C atoms of dpa and two of the three bridging sulfide ligands of the cluster. This reaction has been thoroughly described in the literature, and it is known to proceed with relatively low barriers, as shown in Figure 2.^33^ At this point, interaction between intermediate **I** and H_2_ results in the cleavage of the latter, together with the formation of an S–H and a C–H bonds. With a free energy barrier of 35.8 kcal·mol^–1^, **TS**_**II**_ represents the computed rate-determining step of the whole process. Unfortunately, such barrier does not agree with the mild experimental conditions required for the catalytic process and therefore suggests that it is not the operating mechanism. Further comparison of experimental and computed selectivities points toward the same conclusion. From **II**, the (*Z*)-isomer can be released via **TS**_**II-***cis*_. Alternatively, **II** can rearrange into **III** through **TS**_**III**_ and afford (*E*)-isomer. Alkene selectivity according to this mechanism is determined by the energy difference between **TS**_**II-***cis*_ and **TS**_**III**_, which feature free energy barriers of 5.0 and 3.0 kcal·mol^–1^, respectively. Based on these values, (*E*)-stilbene should be the major product (computed enantiomeric excess (*ee*): 89.9), while experimentally this isomer is not observed. Hence, in light of these results this mechanism can be discarded.

### Mechanism via H_2_ Addition to the Cluster

**Figure 3:**
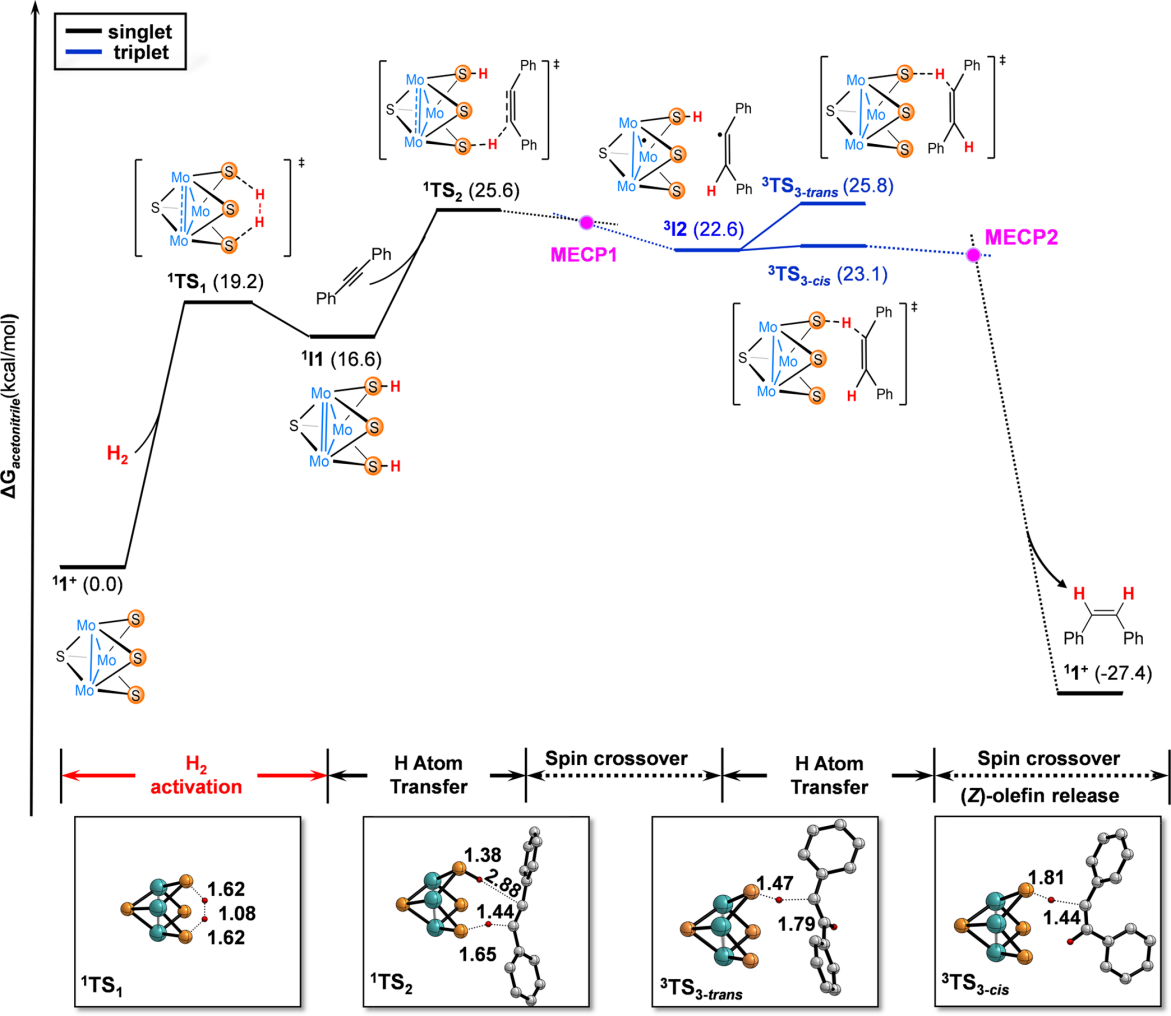
Gibbs energy profile for the semihydrogenation of dpa through the bis(hydrosulfido)-mediated mechanism. Free energy profiles for the singlet and triplet states are given in kcal mol^–1^, quoted relative to **1**^+^ + dpa + H_2_. Ligands and hydrogen atoms have been omitted for the sake of clarity (except vinylic hydrogens). Selected bond distances are given in Å.

At this point, we moved into the computational analysis of an alternative sulfur-mediated pathway reminiscent to that proposed for the hydrogenation of azobenzene.^31,34^ This involves (i) hydrogen activation by the sulfide centers of the cluster; (ii) direct interaction with the substrate; and (iii) release of the hydrogenated molecule, represented in Figure 3.

The initial step of the catalytic cycle entails the homolytic cleavage of H_2_ to generate a bis(hydrosulfido) species (see Figure 3, H_2_ activation). This step is computed to take place via ^**1**^**TS**_**1**_ with a free energy barrier of 19.2 kcal·mol^–1^, leading to intermediate [Mo_3_(μ_3_-S)(μ–S-H)_2_(μ-S)(ImNH_2_)_3_]^+^ (^1^**I1**). The formation of this species is endergonic by 16.6 kcal·mol^–1^, and inspection of its structure shows two identical S–H bonds resulting from the homolytic H_2_ activation. This addition triggers the shortening of one Mo–Mo bond—from 2.787 to 2.677 Å—indicative of an electronic rearrangement within the system whereby the Mo centers undergo a redox process from Mo_3_^IV^ to Mo^IV^Mo_2_^III^.^28,31^ The structural parameters of ^1^**I1** suggest the presence of eight CSE in the Mo_3_S_4_ unit, which prompted us to corroborate the assigned closed-shell singlet configuration. For that purpose, we optimized the **I1** intermediate as an open-shell singlet and as a triplet (see Table S3). While the open-shell singlet converged to the closed-shell singlet, the triplet state lied 8.3 kcal·mol^–1^ above the singlet state. Incidentally, this mechanism shares common features with the reductive activation pathway whereby H_2_ dissociation results in 2H^+^ and 2e^–^.^30^

The subsequent step involves the transfer of H atoms to dpa to afford the corresponding alkene. All attempts to transfer both H atoms via a synchronous transition step were unsuccessful and therefore alternative pathways based on stepwise processes were considered. As shown in Figure 3, the transfer of the first H atom into dpa (^**1**^**TS**_**2**_) features a relative free energy of 25.6 kcal·mol^–1^, the rate-determining step of the whole process. This value is in good agreement with the experimental requirements of moderate pressure (20 bar) and temperature (70 °C) to obtain the product. In fact, a similar tendency was observed in earlier studies on the hydrogenation of unsaturated N=N bonds catalyzed by Mo_3_S_4_ clusters, whereby the hydrogen transfer also represented the rate-determining step.^31^

From a structural perspective, ^**1**^**TS**_**2**_ features the expected H atom that is being transferred halfway between the sulfide ligand to which it was bound and the alkyne C atom to which it will bind. The product of ^**1**^**TS**_**2**_, labeled as ^**1**^**I2**, features a relative free energy of 24.8 kcal mol^–1^ (see Table S6) and consists of a weakly bound adduct between the half-hydrogenated dpa molecule and the cluster with a single S–H group. A key question at this point is whether this process is best described as the transfer of a proton, a hydrogen atom, or a hydride ligand. For that purpose, the two moieties within **I2** were computed separately considering each of the three possibilities (Table S2). The relative free energy (to the reactants) of these two species when a hydrogen atom transfer (HAT) process occurs is 23.2 kcal mol^–1^. In contrast, the calculated free energies for these species show significantly higher values, 43.2 and 61.6 kcal·mol^–1^, for proton and hydride transfers, respectively. These findings claim that this process is better framed within a hydrogen atom transfer, not a proton or a hydride transfer.^35^

**Figure 4:**
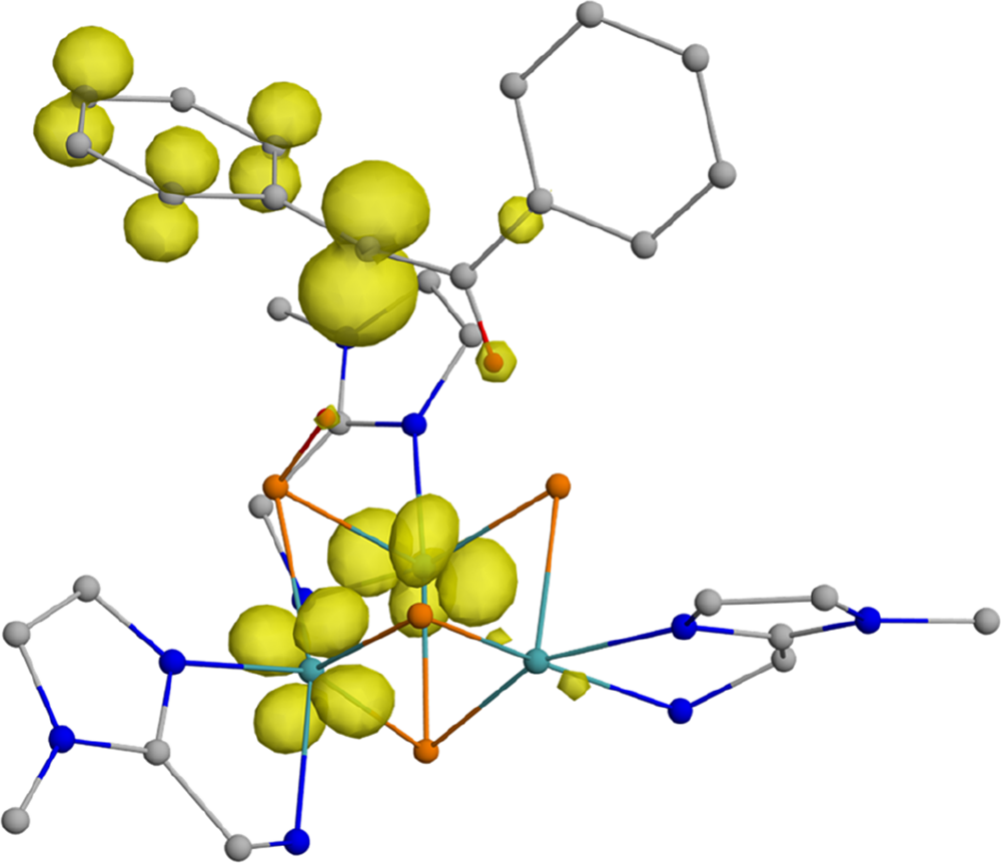
Spin density map of intermediate ^**3**^**I2** calculated at the UBP86/6-31G(d,p) level. The isovalue was arbitrarily chosen to be 0.008 au. Hydrogen atoms were omitted for clarity except those contained in the substrate and in the S–H moieties. Color code: Mo(cyan), S(orange), N (blue), C(gray), H (red).

Considering the above results, we decided to take a closer look at the electronic structure of **I2**.Computation of this adduct in the triplet state (^**3**^**I2**) shows a stabilization of 2.2 kcal·mol^–1^ with respect to ^**1**^**I2** (22.6 vs 24.8 kcal·mol^–1^), suggesting the formation of species with unpaired electrons. Single point calculation of the ^**3**^**I2** structure as an open-shell singlet results in a value of 3.8 kcal·mol^–1^ above that of ^**3**^**I2** (in terms of electronic energies), confirming its nature as a triplet state (Table S3). Analysis of the distances in the optimized ^**3**^**I2** reveals a slight elongation (ca. 0.059 Å) of one of the Mo–Mo bonds while the others recovered its initial distances (Figure S12). As we mentioned before, a similar tendency in the intermetallic distances is found in the Mo_2_^IV^Mo^III^ seven CSE species.^27^ Based on these structural parameters, we envision that one unpaired electron is located in the Mo_3_S_4_ unit, while the other lies in the organic substrate. This assumption is confirmed by the spin density map of ^**3**^**I2** depicted in Figure 4.

The spin density in the organic moiety is mostly localized on the non-hydrogenated carbon atom, with a minor portion being distributed across the adjacent benzene ring. The rest of the spin density is found on the two Mo-centers bridged by the remaining S–H group (Table S4).

A minimum energy crossing point (MECP1) was computed between ^**1**^**TS2** and ^**3**^**I2**, confirming that the first transfer occurs with a spin change. This structure (MECP1) at the BS2 level appears on the potential energy surface 4.6 kcal mol^–1^ below ^**1**^**TS2**, so the spin crossover is expected to be barrierless. Then, intermediate ^**3**^**I2** releases either (*Z*) or (*E*)-stilbene through a second H atom transfer and regenerates the initial **1**^+^ species. The transition states for these processes, namely, ^**3**^**TS**_**3-***cis*_ and ^**3**^**TS**_**3-***trans*_, have relative free energies of 23.1 and 25.8 kcal mol^–1^, respectively. Note that, based on the previous computations, we expect that their counterparts in the singlet potential energy surface could not be optimized. Comparison of both structures indicates that the higher relative free energy of ^**3**^**TS**_**3-***trans*_ is likely due to the steric hindrance as a result of the interaction between the phenyl rings of the alkyne and the cluster. The energy difference between the two transition states is computed to lead to an enantiomeric excess of 96.3 in favor to the (*Z*)-stilbene in line with the experimental observation.^29^ Moreover, ^**3**^**TS**_**3-***cis*_ is only 0.5 kcal mol^–1^ above adduct ^**3**^**I2**, so this second HAT is expected to occur immediately after the formation of the latter species, releasing (*Z*)-stilbene. A minimum energy crossing point (MECP2) was located after ^**3**^**TS**_**3-***cis*_ in order to recover the singlet state and regenerate catalyst **1**^**+**^.

**Scheme 1:**
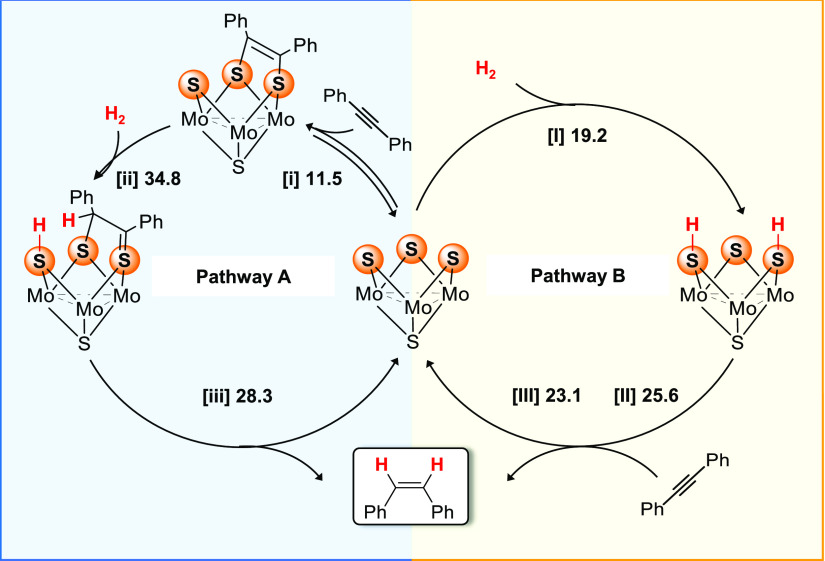
Schematic Representation of the Proposed Catalytic Cycles for the Semihydrogenation of dpa Catalyzed by **1**^**+**^ Relative free energies for the TSs are given in kcal mol^–1^.

According to the DFT studies, the bis(hydrosulfido)-mediated mechanism is therefore consistent with the thermodynamic preference and selectivity control. However, the experimental results confirmed the existence of an equilibrium with the cycloaddition product [Mo_3_S_4_Cl_3_(ImNH_2_)_3_(dpa)]^+^ along the reaction course. In order to obtain a better understanding of the plausible mechanism, both catalytic cycles are summarized in Scheme 1. The first step of the catalytic cycles entails the formation of the dithiolene (pathway A) vs bis(hydrosulfido) (pathway B) species. It should be noted that the formation of the dithiolene adduct (pathway A) was computed to feature a relatively low free energy barrier (11.5 kcal·mol^–1^, pathway A), with the step being slightly exergonic by −1.0 kcal mol^–1^. This supports the experimentally observed equilibrium between the trinuclear cluster and the dithiolene adduct.^29^

The dynamic character of the system plays a critical role in the mechanism since both species, the cluster and the dithiolene complex, are in solution, leading to the interconnection of the two catalytic cycles. From this situation, the formation of the hydrosulfido S–H bonds ([I], pathway B) on **1**^**+**^ has the lowest computed barrier for the H_2_ activation (Δ*G*^‡^ = 19.2 kcal·mol^–1^). In contrast, the cleavage of the hydrogen molecule via interaction with the cycloaddition product ([ii], pathway A) is too energy-demanding to take place under these reaction conditions (Δ*G*^‡^ = 34.8 kcal·mol^–1^). Considering all of the gathered information, we believe that an equilibrium between the cluster and the dithiolene complex is initially established, which supports the initial detection of the aforementioned complex. However, hydrogenation of the dithiolene adduct is too energy-demanding in comparison with the activation of H_2_ at **1**^**+**^, and therefore the system is forced to continue through pathway B (Scheme 1).

### Experimental Investigations

**Scheme 2:**
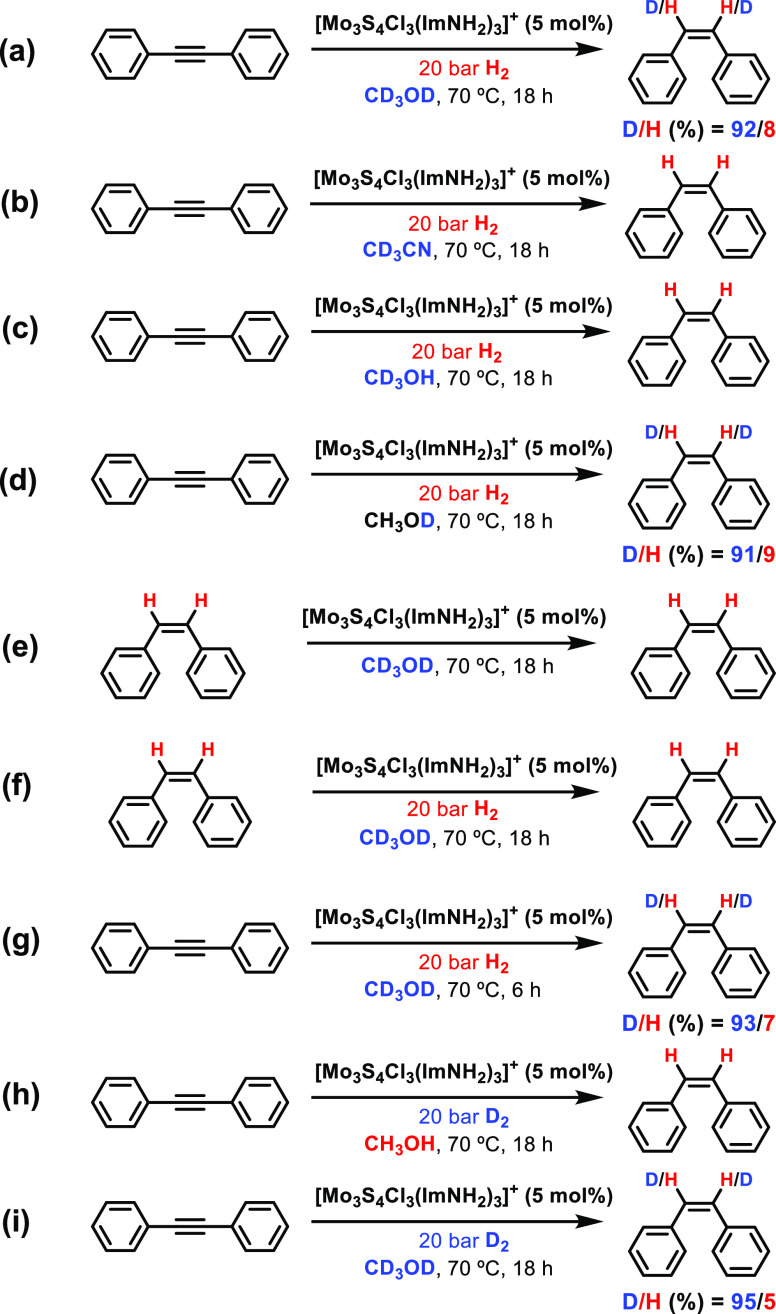
Deuterium Labeling Experiments upon Different Deuterated Solvents (a, b), Deuteration Position (CH_3_OD and CD_3_OH) (c, d), Lower Reaction Time (g) and Using D_2_ as the Reducing Agent (h, i), and Deuteration of (Z)-Stilbene (e, f)

To further verify the proposed mechanism, a series of experiments aimed to obtain additional mechanistic evidence were conducted (see Experimental Section for more details on the procedure). The proposed mechanism involves the participation of hydrosulfido moieties in the catalytic cycle, which possess an acidic character,^36^ as revealed by the measured p*K*_a_ values of some bimetallic hydrosulfide complexes.^37^ Regarding trinuclear molybdenum sulfide clusters, a value of 4.1 (calculated p*k*_*a*_)^31^ was obtained, which follows the same tendency as its dinuclear analogues. Based on these considerations, we envisioned that the introduction of a solvent with an acidic deuterium (D) atom with a higher p*K*_*a*_ could result in the exchange of the S–H groups to S–D at a faster rate than the H transfer to the alkyne, thus labeling the vinylic positions. The results of deuterium labeling experiments are shown in Scheme 2, where the deuterium content has been obtained by integration of the vinylic and aromatic CH signals in the ^1^H NMR spectra (Figures S1–S11).

The results clearly indicate that deuterium was highly incorporated (deuteration degree: >90%) into both olefinic positions when CD_3_OD or CH_3_OD were used as solvent (Scheme 2a,d, Figure S2 and S4). The deuteration degree was not quantitative due to water traces in the solvent, which also exchange with the S–H/D moieties. In contrast, no signal of deuteration was observed when using CD_3_CN or CD_3_OH as solvent (Scheme 2b,c, Figure S1 and S3), thus showing that the source of deuterium atoms in (*Z*)-stilbene are those in the alcoholic group of methanol.

Additional experiments ruled out the possibility that the deuterium atoms in (*Z*)-stilbene were a result of a hydrogen isotope exchange (HIE) or reductive deuteration processes of the alkyne.^38^ Direct C–H activation through a HIE process rendering H/D exchange^39^ can be discarded by the absence of deuterated product when (*Z*)-stilbene is dissolved in CD_3_OD (Scheme 2e, Figure S5). The same tendency was observed including H_2_ gas during the catalytic reaction (Scheme 2f, Figure S6). These results preclude a HIE reaction after releasing (*Z*)-stilbene from the semihydrogenation reaction.^38^ In fact, when the reaction was performed at shorter times, comparative deuterium contents to those obtained after 18 h were observed (Scheme 2g, Figure S7). The possibility of deuterium atoms being incorporated into dpa by interaction with deuterated or semideuterated hydrogen, either added as D_2_ or resulting from H_2_ exchange with S-D groups, can be also ruled out on the basis of Scheme 2e–i (Figures S8 and S9). The absence of deuterated (*Z*)-stilbene in Scheme 2h can be rationalized considering the very rapid exchange between the S–D moieties just formed and the O–H groups of the methanol that would take place before the alkyne approach to the hydrogenated cluster. Incidentally, the presence of water traces in CD_3_OD was confirmed when deuterium gas is used as reductant (Scheme 2i, Figure S9) because 5% of the hydrogenated product was observed.

Thus, deuterium labeling experiments provide support for the formation of intermediates containing S–H groups. In addition, an important difference between the computed mechanisms above is that only one S–H group is formed in the dithiolene-mediated mechanism (Scheme 1, pathway A) whereas two S–H groups are formed in the mechanism involving initial H_2_ activation (Scheme 1, pathway B). Thus, both mechanisms are expected to differ in the number of C–H bonds of (*Z*)-stilbene that will be deuterated, one in the dithiolene mechanism and two in the mechanism involving initial H_2_ activation. The experimental results showing that both C–H groups in (*Z*)-stilbene are deuterated clearly favor the bis(hydrosulfide)-mediated mechanism, which includes the formation of two S–H groups.

As pointed out above, we have previously reported that the related [Mo_3_S_4_Cl_3_(dmen)_3_]^+^ cluster also catalyzes the semihydrogenation of dpa,^28^ although with a lower efficiency and selectivity, and proposed a mechanism similar to that depicted in Figure 2 for **1**^+^. Given the present results, which clearly point toward a different reaction mechanism, we decided to carry out deuteration experiments also for the [Mo_3_S_4_Cl_3_(dmen)_3_]^+^ cluster. Experiments in CH_3_OH indicate that hydrogenation occurs with a yield of 24% using 90 °C, 40 bar H_2_, 12 mol % of catalyst, and 65 h, thus confirming the lower catalytic activity of this cluster (Figure S10). As observed in CH_3_CN, a mixture of (*Z*) and (*E*)-isomers in an *ca*. 6:1 ratio is formed. When the experiments with [Mo_3_S_4_Cl_3_(dmen)_3_]^+^ are carried out in CD_3_OD, significant deuteration (78%) of (*Z*)-stilbene is observed (Figure S11). Unfortunately, the degree of deuteration of the (*E*)-isomer could not be determined because the signal for its vinylic protons overlaps with those for the aromatic protons of the (*Z*)-isomer, which is the major product. Yet, the 78% deuteration degree for (*Z*)-stilbene is halfway between those expected for the previously reported mechanism and the one proposed here for **1**^+^, thus suggesting that both mechanisms are competitive in the case of the [Mo_3_S_4_Cl_3_(dmen)_3_]^+^ cluster. At this point, we calculated the energy of the rate-determining transition state with our new mechanistic proposal (^**1**^**TS**_**2**_, Figure 3) for the [Mo_3_S_4_Cl_3_(dmen)_3_]^+^ cluster (Table S7). The free energy value of ^**1**^**TS**_**2dmen**_ is 33.1 kcal mol^–1^, that is 7.5 kcal mol^–1^ above the calculated ^**1**^**TS**_**2**_ (25.6 kcal·mol^–1^) for **1**^+^. The energy of **TS**_**IIdmen**_ was previously reported to be of 42.0 kcal mol^–1^,^28^ also significantly larger than for **1**^+^ (see **TS**_**II**_, Figure 2), thus showing that independently of the actual reaction mechanism operating for the dmen cluster, its reaction is much slower than that of **1**^+^. However, the difference between the energies corresponding to both mechanisms for [Mo_3_S_4_Cl_3_(dmen)_3_]^+^ is 8.9 kcal mol^–1^, very close to the corresponding value for **1**^**+**^ (9.2 kcal mol^–1^), which indicates that the mechanism in Figure 3 should be also preferred in the case of [Mo_3_S_4_Cl_3_(dmen)_3_]^+^. With these results, we cannot give a satisfactory explanation to the reasons leading to the operation of both mechanisms in the case of this dmen cluster, which are probably related to subtle aspects not captured by the present calculations.

## Conclusions

Computational and experimental studies on the selective semihydrogenation of dpa catalyzed by cluster [Mo_3_S_4_Cl_3_(ImNH_2_)_3_]^+^ (**1**^+^) reveals that the previously proposed mechanism for the related [Mo_3_S_4_Cl_3_(dmen)_3_]^+^ cluster, in which there is initial cycloaddition of the alkyne to two bridging S, is unable to explain the major experimental findings for **1**^+^: milder reaction conditions; higher selectivity toward the (*Z*)-isomer; and complete deuteration of the reaction product. In contrast, a thorough exploration allowed us to propose a novel mechanism reminiscent of that recently reported for the hydrogenation of azobenzene, which accounts for the experimental results. The first step entails the H_2_ activation in the sulfur units that generates the [Mo_3_(μ_3_-S)(μ–S-H)_2_(μ-S)]^+^ intermediate followed by two consecutive hydrogen atom transfers (HAT) from the bis(hydrosulfido) species to the alkyne. The product of the first HAT can be formally described as a radical pair with one of the unpaired electrons in the Mo_3_S_4_ moiety and the other in the semihydrogenated alkyne. As a result of the unpaired electrons that the system features, a spin crossover process between the singlet and the triplet spin states is necessary along the first HAT. In the final step, the radical pair species undergo a quasi-barrierless second HAT with another concomitant spin crossover, from triplet to singlet state, to release the (*Z*)-stilbene and to regenerate the **1**^**+**^ cluster. The computations indicate that the unobserved (*E*)-stilbene could also be formed from this radical pair; however, steric effects result in an energy barrier 2.7 kcal mol^–1^ higher, which is in line with the exclusive formation of (*Z*)-stilbene. Remarkably, the deuteration of the vinylic positions in the (*Z*)-stilbene was achieved using H_2_ and nonexpensive CD_3_OD, thus opening new avenues for the synthesis of labeled molecules using molybdenum sulfide clusters as catalysts in reductive deuteration reactions.

## Experimental Section

### General Considerations

The [Mo_3_S_4_Cl_3_(ImNH_2_)_3_]BF_4_ and [Mo_3_S_4_Cl_3_(dmen)_3_]BF_4_ catalysts were prepared according to the published procedure.^24,29^ All other reagents were obtained from commercial sources and used as received.

^1^H NMR spectra were recorded on a Bruker Avance III HD 400 MHz spectrometer using *d*_6_-dimethyl sulfoxide (DMSO) as solvent. Gas chromatography analysis were performed on an Agilent 7820A GC System equipped with a FID and a capillary column Agilent (HP-5, 30 m x 0.32 mm × 0.25 mm).

### General Procedure for the Catalytic Semihydrogenation of Diphenylacetylene

A 4 mL glass vial containing a stirring bar was sequentially charged with the corresponding molybdenum catalyst (4.5 mg, 0.005 mmol of [Mo_3_S_4_Cl_3_(ImNH_2_)_3_]BF_4_]), diphenylacetylene (18.0 mg, 0.1 mmol), n-hexadecane (15 μL; added as an internal standard), and 2 mL of the corresponding solvent (CH_3_CN, CD_3_CN, CD_3_OD, CH_3_OD, CD_3_OH). Afterward, the reaction vial was capped with a screw cap containing a septum with a needle and set in the alloy plate, which was then placed in a 300 mL autoclave. The sealed autoclave was purged three times with 30 bar of hydrogen before setting the pressure at 20 bar. Then, it was placed into an aluminum block, which was preheated at 70 °C. After 18 h, the autoclave was cooled to room temperature, and the hydrogen was released. Ethyl acetate (2 mL) was then added, and a sample was analyzed by GC. To determine the deuterium content, the reaction mixture was taken to dryness via rotatory evaporation and solved in a deuterated solvent (*d*_*6*_-DMSO).

### Computational Details

All the density functional theory (DFT) calculations were performed with Gaussian 09 (Revision D.01).^40^ Geometry optimizations were carried out at the BP86/BS1 level,^41,42^ where Mo and S atoms were described using the SDD relativistic ECP and associated basis set,^43^ added polarization functions for the latter (ζ = 0.503),^44^ and the remaining atoms were described with the 6-31G(d,p) basis set.^45,46^ Solvent effects (acetonitrile, ε = 35.688) were included self-consistently in these optimizations through the PCM method.^47,48^ All stationary points were characterized at this level of theory by analytical frequency calculations as either minima (all positive eigenvalues) or transition states (one imaginary eigenvalue), while intrinsic reaction coordinate (IRC) calculations and subsequent geometry optimizations were used to confirm the minima linked by each transition state. The frequency calculations were also used to obtain the thermochemistry corrections (zero-point, thermal, and entropic energies) at the experimental temperature (343.15 K) and at the standard 1 atm pressure, on the basis of the IGRRHO (ideal gas/rigid rotor/harmonic oscillator) approach. However, these pressures and temperatures do not correspond to the 1 M concentration of the standard state used for species reacting in solution. Therefore, corrections (2.275 kcal·mol^–1^) were applied to all Gibbs values to change the standard state to 1 M at 343.15 K. This correction has been calculated using the formula RTln *V*_m_= 2.275 kcal·mol^–1^, where *V*_m_ = 28.1 L·mol^–1^ and corresponds to the molar volume of an ideal gas at 1 atm and 343.15 K.

Improved energetic values were obtained by performing single-point energy calculations with a larger basis set system (BS2), also including solvent effects through the PCM method.^47,48^ BS2 differs from BS1 in the employment of the 6-311+G(2d,2p) to describe Cl, C, N, O, and H atoms. In addition, single-point dispersion corrections were computed using Grimme’s D3 (zero damping) parameter set.^49^ Thus, the Gibbs energies in acetonitrile(*G*_acetonitrile_) shown in the text were obtained adding to the potential energies in acetonitrile calculated at BP86/BS2, the Gibbs contribution at the BP86/BS1 level, the dispersion correction, and the standard state correction. All of the above energetic values are provided in Table S6.

The minimum energy crossing points (MECPs) were located using Harvey’s algorithm combined with Gaussian 09.^50^ The three-dimensional (3D) structures were depicted using CYLview software and VESTA for the spin density map.^51,52^

## Supporting Information Available

NMR spectra, DFT results, and Cartesian coordinates for all optimized systems (PDF)

## ABBREVIATIONS

ImNH_2_: 1-methyl-1*H*-imidazol-2-ylmethanamine.
