# Supplementary material for: Spin-Crossing in the (Z)-Selective Alkyne Semihydrogenation Mechanism Catalyzed by Mo3S4 Clusters: A Density Functional Theory Exploration
Source: Inorg Chem. 2024 Jan 4;63(2):1000–9. doi: 10.1021/acs.inorgchem.3c03057 (PMC10792605; doi:10.1021/acs.inorgchem.3c03057)
Supplement: Supplementary file 1 — ic3c03057_si_001.pdf [file ic3c03057_si_001.pdf]

Supporting Information

Spin-Crossing in the (*Z*)-Selective Alkyne Semihydrogenation Mechanism Catalyzed by Mo_3S_4 Clusters: A Density Functional Theory Exploration

María Gutiérrez-Blanco,[a] Andrés G. Algarra,[b] Eva Guillamón,[a] M. Jesús Fernández-Trujillo,[b] Mónica Oliva,[a] Manuel G. Basallote,[b] Rosa Llusar[a] and Vicent S. Safont[a]*

[a] Departament de Química Física i Analítica, Universitat Jaume I, Av. Sos Baynat s/n, 12071 Castelló, Spain

b Departamento de Ciencia de los Materiales e Ingeniería Metalúrgica y Química Inorgánica, Instituto de Biomoléculas (INBIO), Facultad de Ciencias, Universidad de Cádiz, Apartado 40, Puerto Real, 11510 Cádiz, Spain.

*Email address of the corresponding author:

Vicent S. Safont : safont@uji.es

Table of contents

1. Mechanistic experiments S2
2. DFT benchmarking study of computed species S8
3. Analysis of the electronic properties of the system – Figure 3 .... S9
4. 3D views of Mo_3S_4 species along the reaction pathway – Figure 3 .... S10
5. Spin densities S11
6. Absolute energies of the computed species S12
7. Cartesian coordinates of the optimized species S13

1. Mechanistic experiments

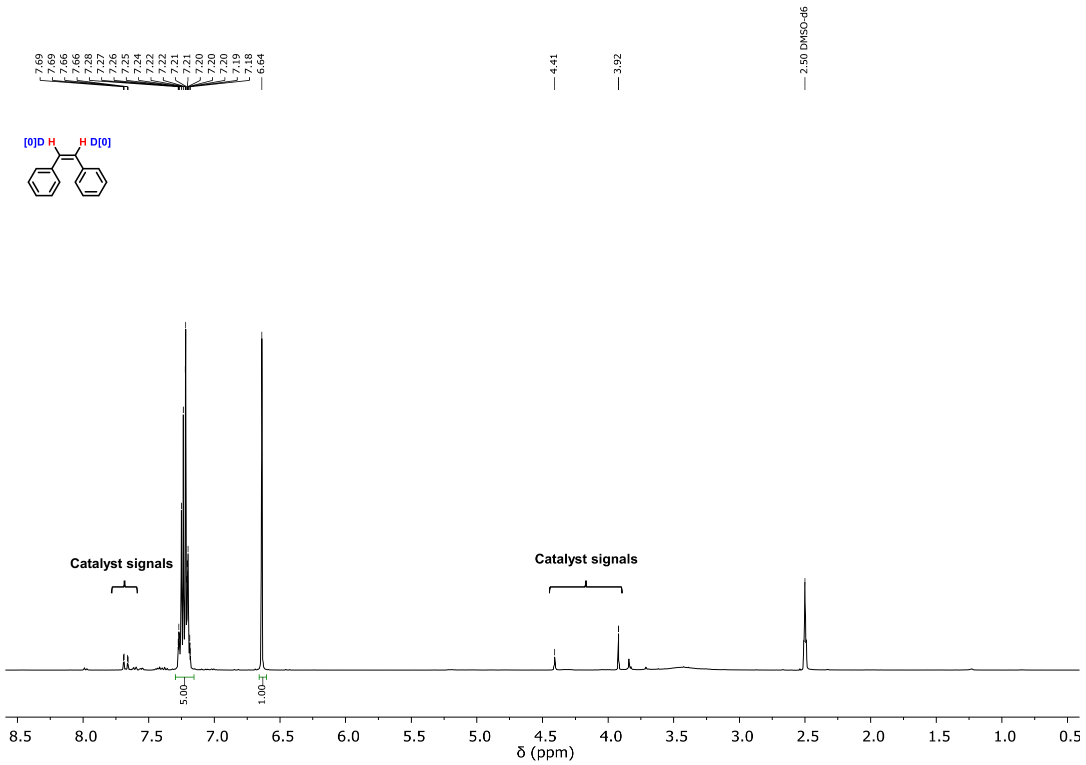

Figure S1. ^{1}H NMR (400 MHz, DMSO, 298 K) spectrum of the reaction mixture after the standard catalytic conditions.

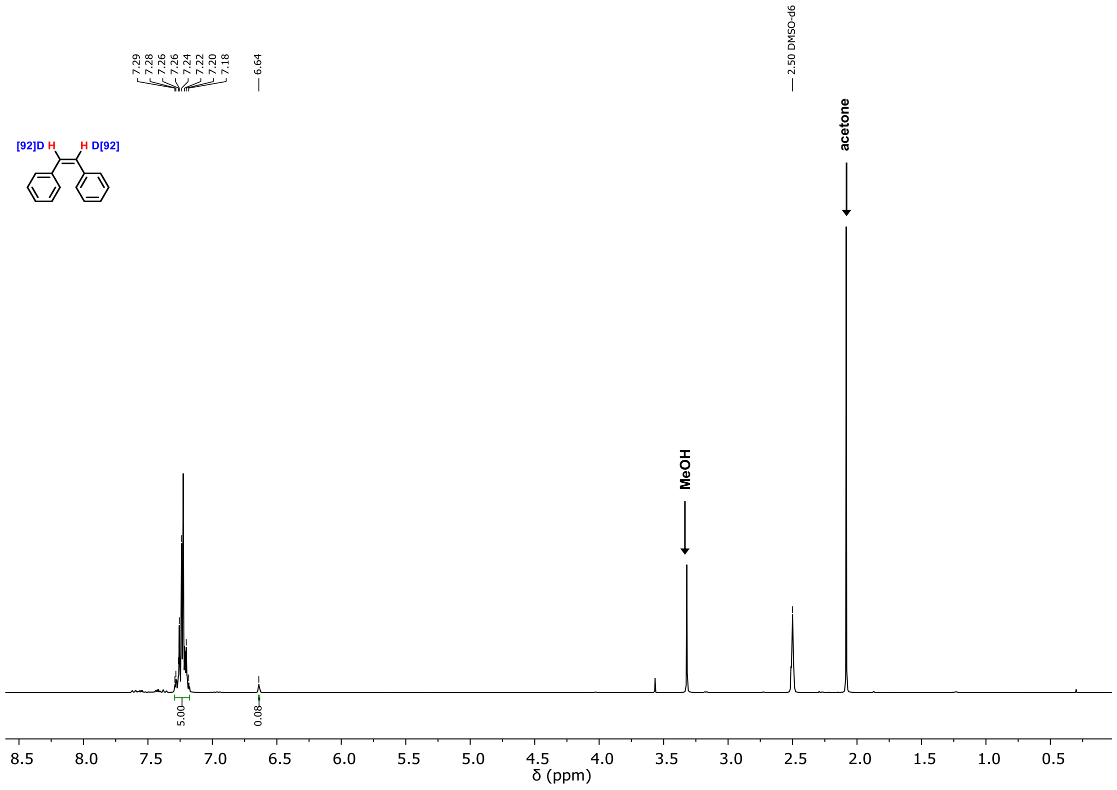

Figure S2. ^{1}H NMR (400 MHz, DMSO, 298 K) spectrum of the reaction mixture after the catalytic protocol using d_4-methanol as solvent.

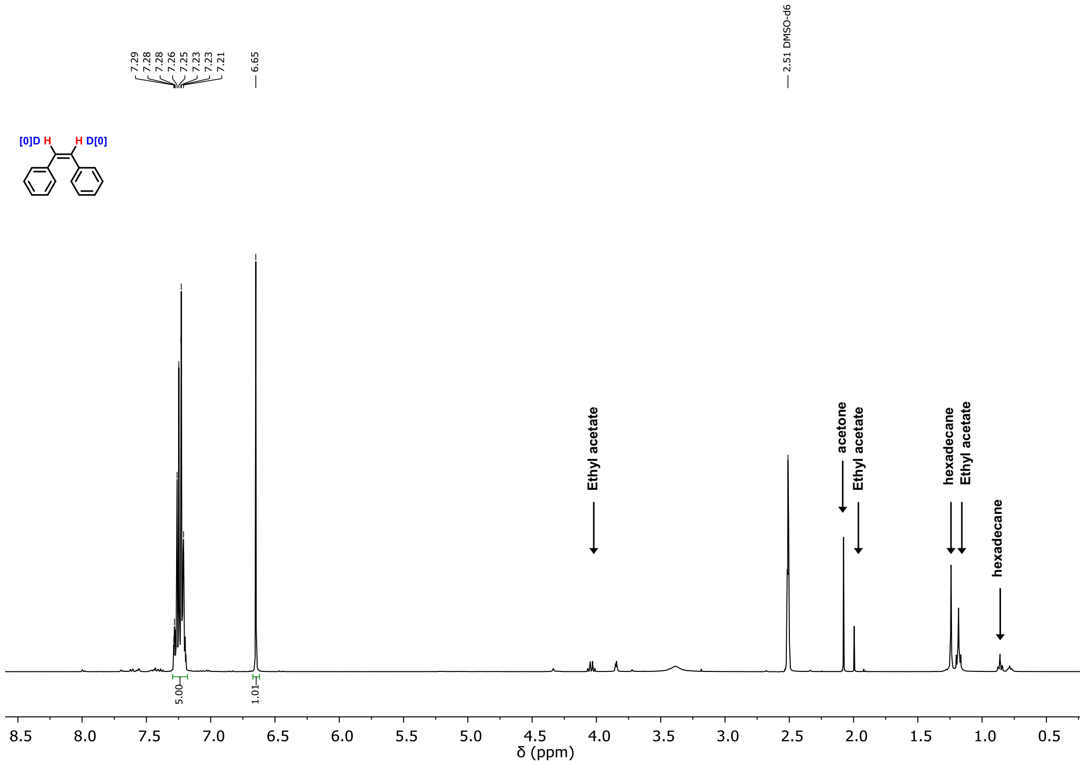

Figure S3. ^{1}H NMR (400 MHz, DMSO, 298 K) spectrum of the reaction mixture after the catalytic protocol using d_3-methanol as solvent.

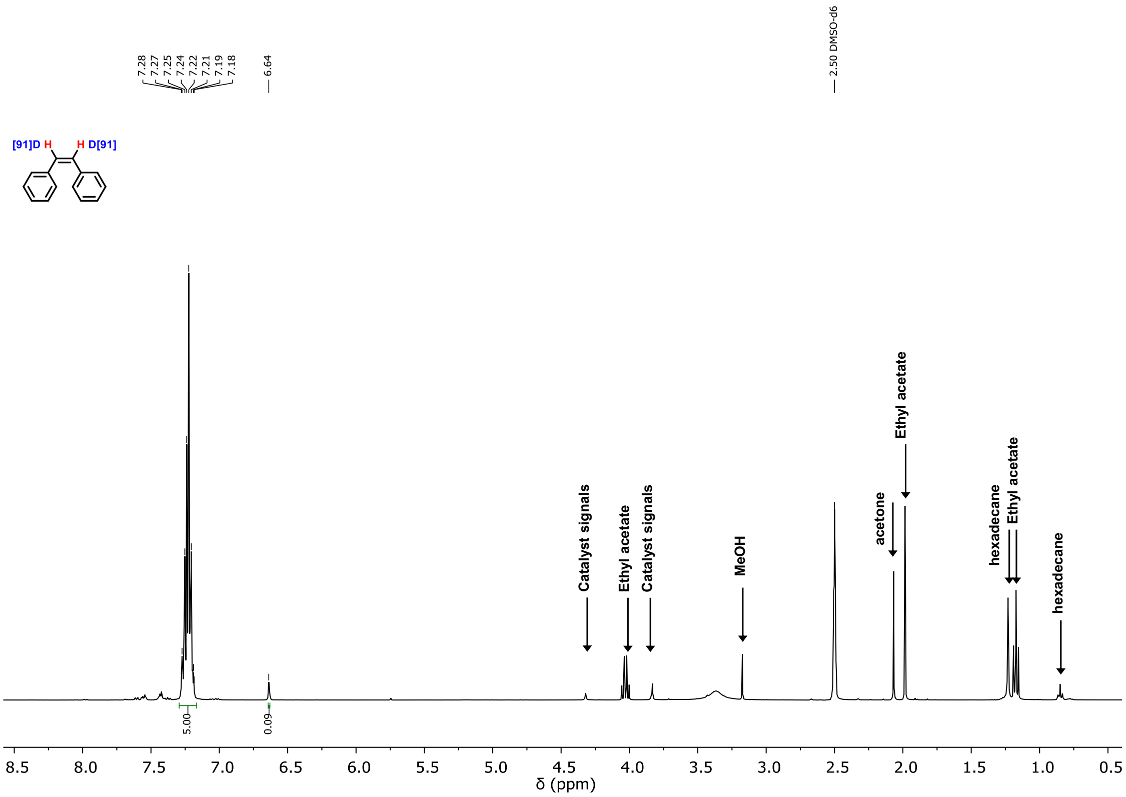

Figure S4. ^{1}H NMR (400 MHz, DMSO, 298 K) spectrum of the reaction mixture after the catalytic protocol using d_1-methanol as solvent.

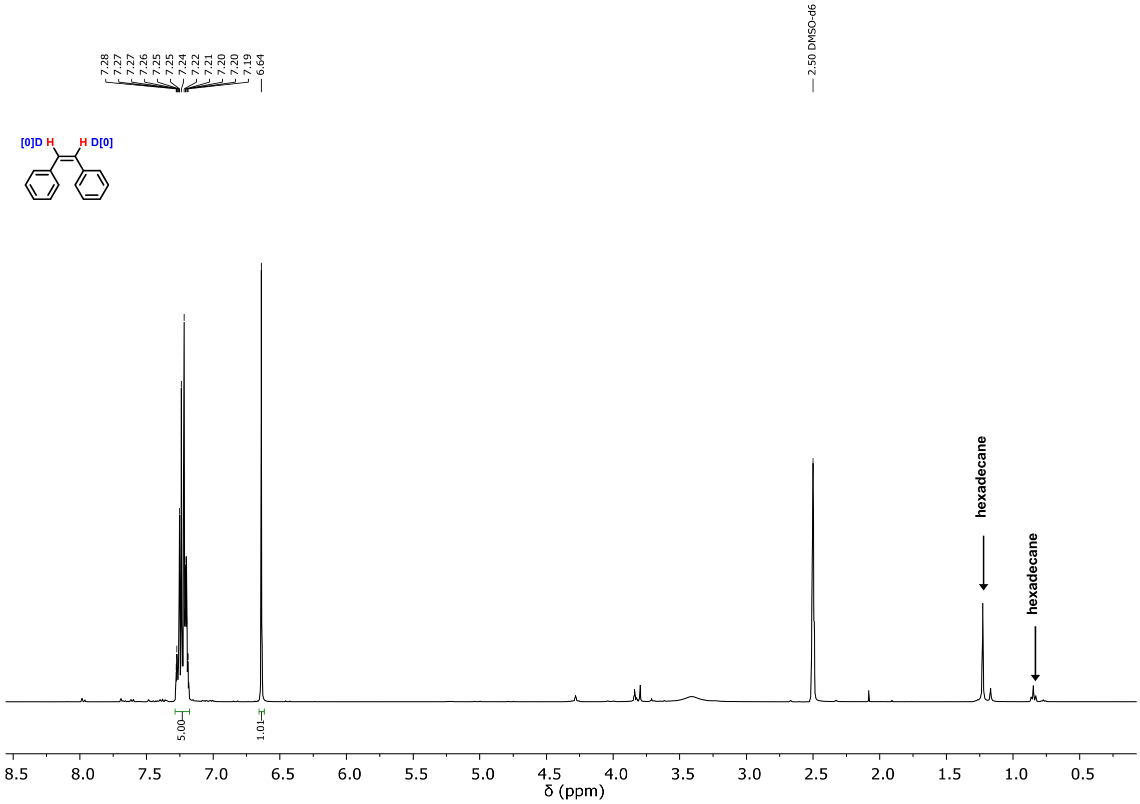

Figure S5. ^{1}H NMR (400 MHz, DMSO, 298 K) spectrum after reacting (*Z*)-stilbene in the standard catalytic conditions in the absence of H_2 using d_4-methanol as solvent.

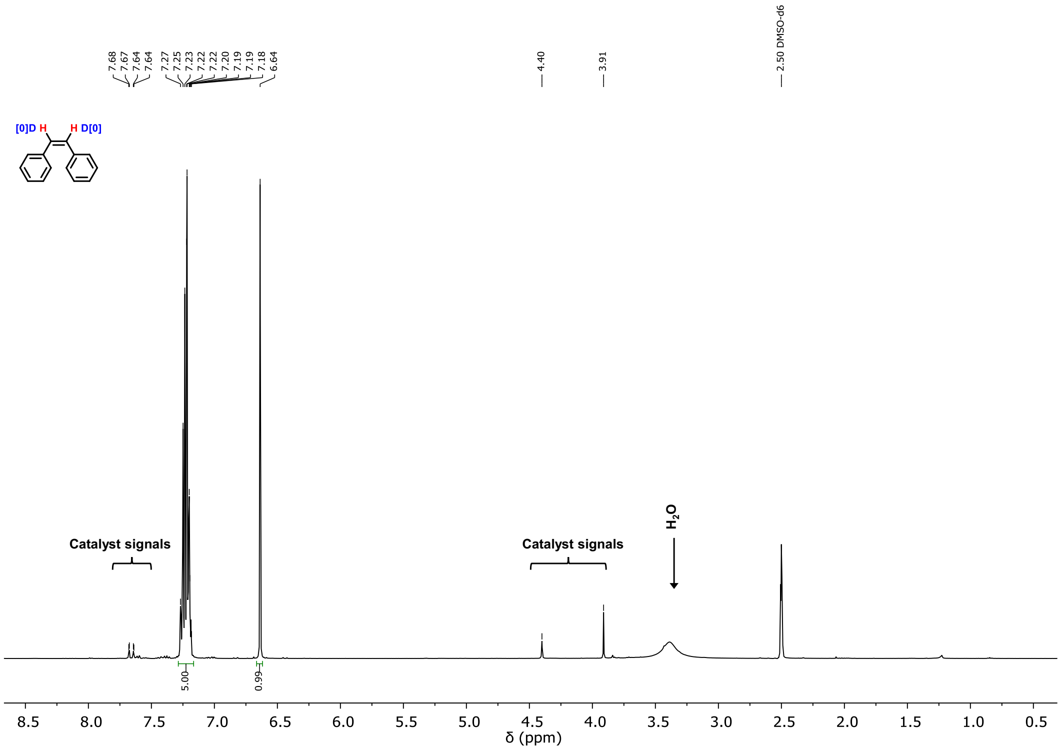

Figure S6. ^{1}H NMR (400 MHz, DMSO, 298 K) spectrum after reacting (*Z*)-stilbene in the standard catalytic conditions using d_4-methanol as solvent.

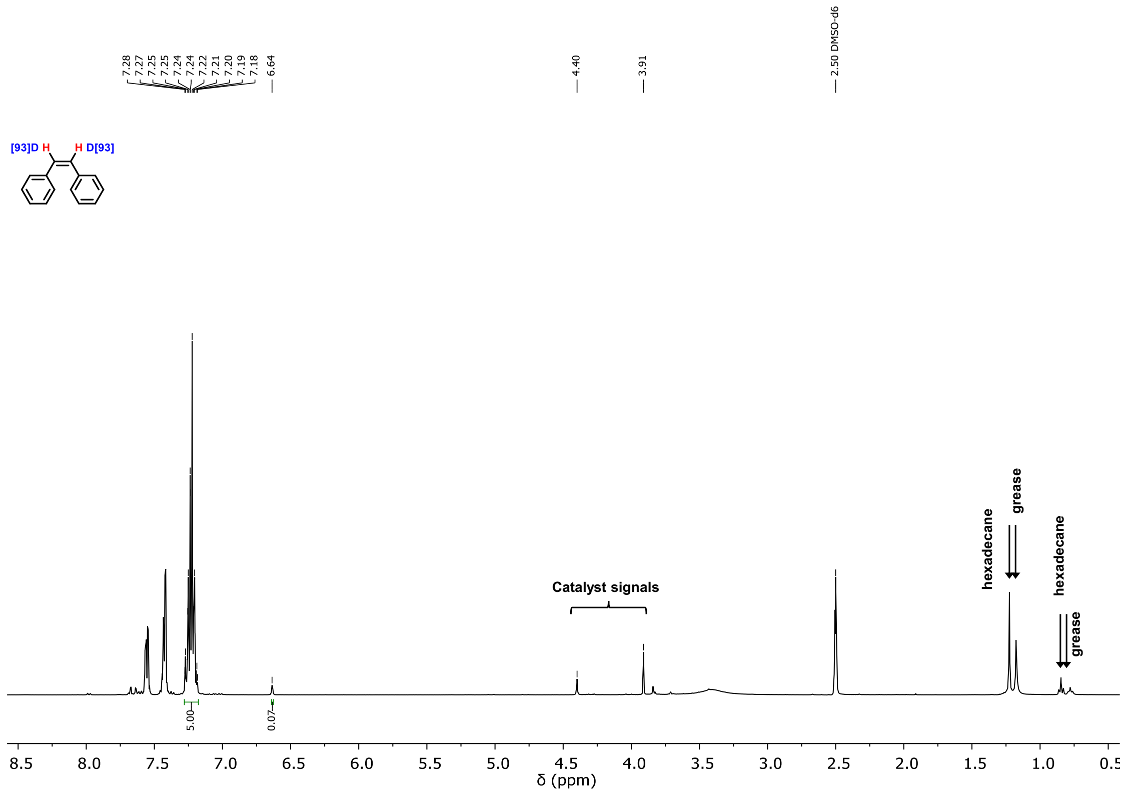

Figure S7. ^{1}H NMR (400 MHz, DMSO, 298 K) spectrum of the reaction mixture after standard catalytic conditions at t = 6 hours.

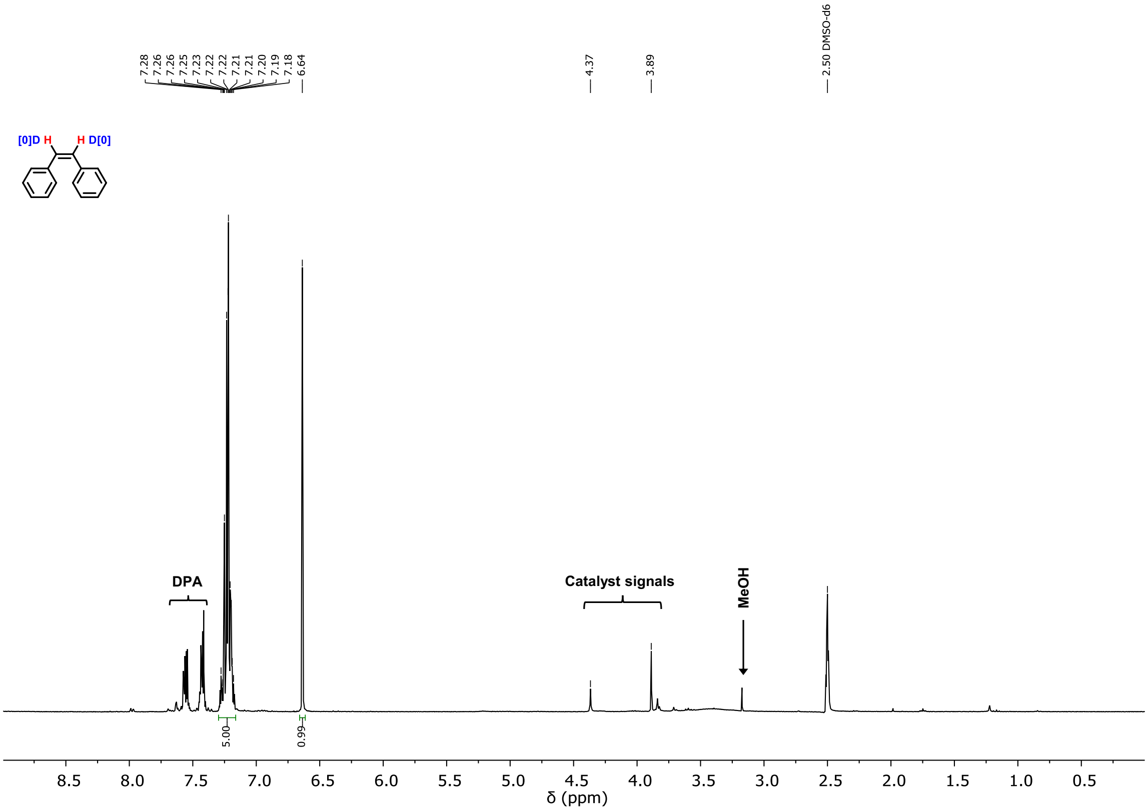

Figure S8. ^{1}H NMR (400 MHz, DMSO, 298 K) spectrum of the reaction mixture after standard catalytic conditions using D_2 gas as reductant.

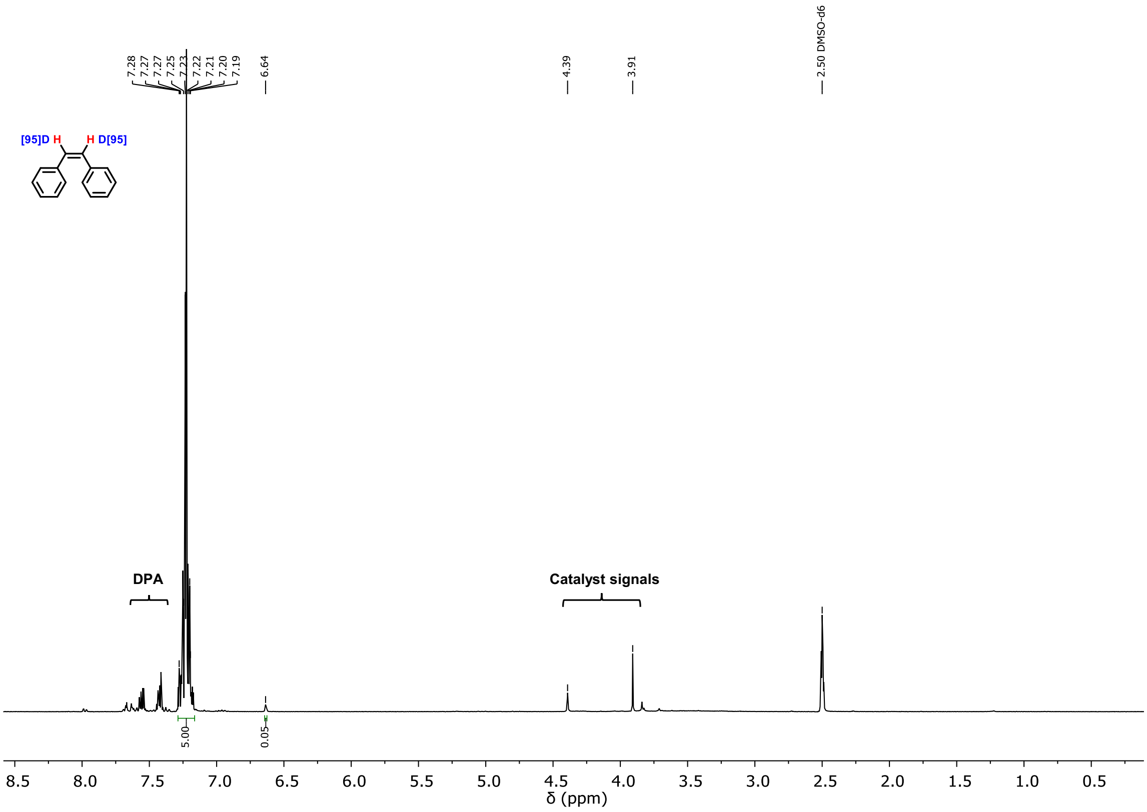

Figure S9. ^{1}H NMR (400 MHz, DMSO, 298 K) spectrum of the reaction mixture after standard catalytic conditions using D_2 gas as reductant and d_4-methanol as solvent.

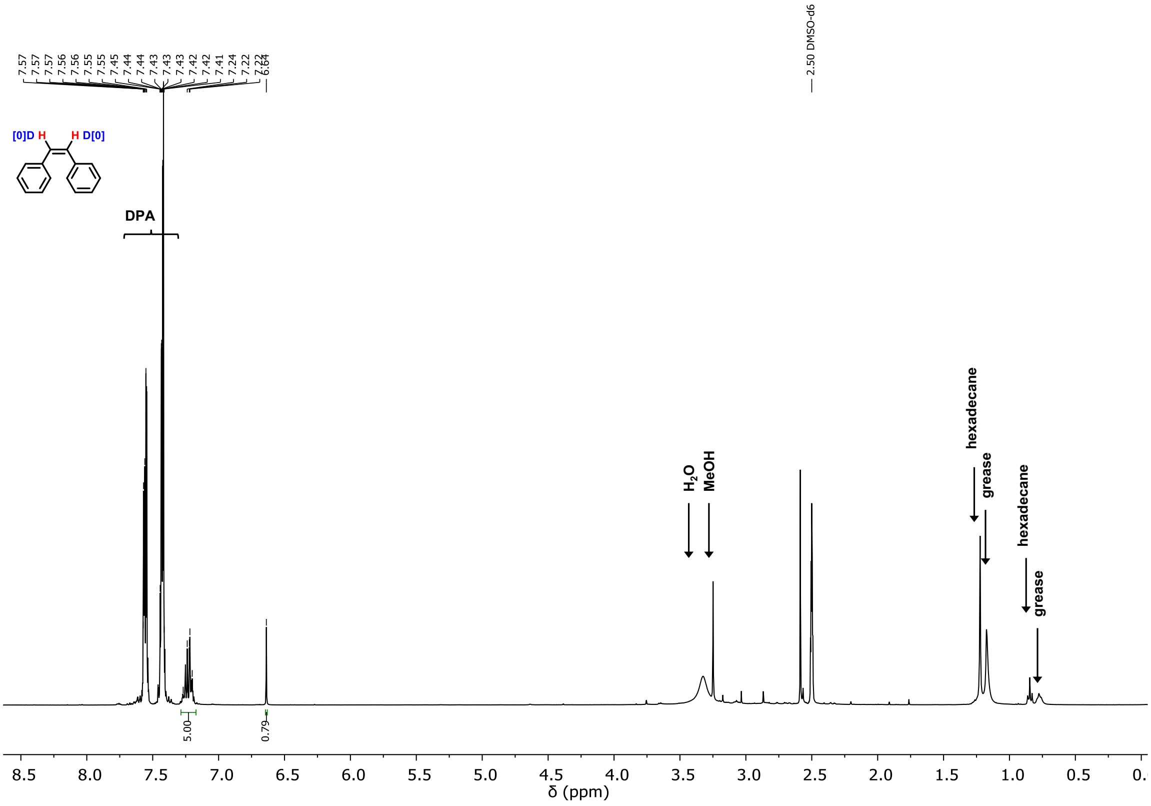

Figure S10. ^{1}H NMR (400 MHz, DMSO, 298 K) spectrum of the reaction mixture catalyzed by $[Mo_3S_4Cl_3(dmen)_3]^+$ complex under the catalytic conditions.

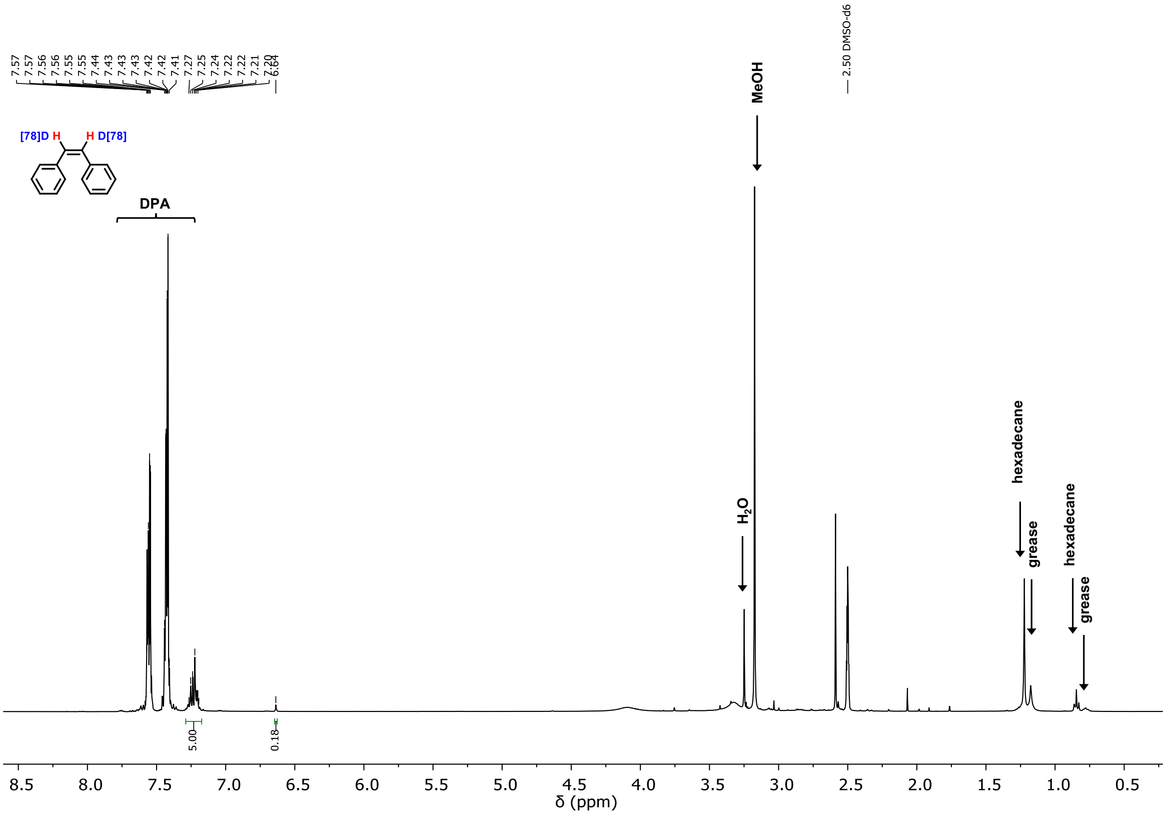

Figure S11. ^{1}H NMR (400 MHz, DMSO, 298 K) spectrum of the reaction mixture catalyzed by $[Mo_3S_4Cl_3(dmen)_3]^{+}$ complex under the catalytic conditions using d_4-methanol as solvent.

2. DFT benchmarking study of computed species

Geometry optimizations were carried out at the BP86/BS1 level and solvent effects (acetonitrile, $\varepsilon = 35.688$) were included self-consistently in these optimizations through the PCM method. In addition, single point calculations with an extended basis set system (BS2), the Gibbs contribution at the BP86/BS1(PCM) level, single-point dispersion corrections using Grimme's D3 parameter set, and the standard state correction, were added to obtain the Gibbs energies reported in the manuscript.

Functional test calculations were carried out via single point energy calculations on the BP86/BS1 optimized geometries using other functionals which are commonly employed (PBE0, B3LYP and M06). These calculations were performed with the appropriate functional using the BS2 basis set and the solvent effects through the PCM method. Moreover, the corrections to go from electronic to free energies were taken from the BP86/BS1(PCM) calculations. Dispersion and standard state corrections were also added.

The results show that the main features of the computed Gibbs energy values remain similar for all the functionals. Despite in all cases the formation of the (*Z*)-stilbene is preferred, the determining step barrier is always significantly higher than for the BP86 functional. Thus, we consider that the selected functional (BP86) is the one that better describes the system in terms of thermodynamics and kinetics. Furthermore, this functional allows us to compare with our previous work on semihydrogenation of alkynes catalyzed by Mo_3S_4 clusters (A. G. Algarra, et al., *ACS Catal.* **2018**, 8, 7346-7350).

Table S1. Free energy values of the proposed mechanism (Figure 3) calculated with different functionals. All values are given in kcal·mol^{-1}.

Stationary point	BP86 [a]	B3LYP	PBE0	M06
**TS_1**	19.1	26.8	19.0	24.7
**I1**	16.6	18.3	12.6	16.1
**$^1TS_2$**	25.6	32.8	30.2	33.6
**$^3I2$**	22.7	21.5	17.1	21.5
**$TS_{3\text{-}cis}$**	23.1	24.6	20.7	27.2
**$TS_{3\text{-}trans}$**	25.8	27.5	24.9	28.8

[a] Values used in the main text.

3. Analysis of the electronic properties of the system – Figure 3

Table S2. Investigation of the character of the transferred hydrogens to the diphenylacetylene in intermediate **I2**. BP86 Electronic energies (E) computed with BS1 and including solvent effects; correction to Gibbs Free energies (G corr.) at the same level of theory; Grimme's D3 dispersion correction; BP86 Electronic energies (E) computed with BS2 and including solvent effects; and final Gibbs Free energies obtained as the sum of the three previous columns. All values are given in Hartrees.

Species	E_{solv}(BS1/PCM)	G corr (BS1/PCM)	D3 corr (0)	E_{solv}(BS2/PCM)	G_{solv}(BS2)
Proton transfer					
Mo_3S_4 unit	-2708.4447	0.3630	-0.1348	-2708.8441	-2708.6159
Substrate	-539.8609	0.1484	-0.0186	-540.0015	-539.8717
Hydrogen atom transfer					
Mo_3S_4 unit	-2708.2988	0.3642	-0.1354	-2708.6958	-2708.4670
Substrate	-540.0317	0.1463	-0.0185	-540.1803	-540.0525
Hydride transfer					
Mo_3S_4 unit	-2708.1171	0.3674	-0.1356	-2708.5129	-2708.2810
Substrate	-540.1367	0.1467	-0.0211	-540.3029	-540.1773

Table S3. Energies of the **I1** and **I2** intermediates in different electronic spin states. BP86 Electronic energies (E) computed with BS1 and including solvent effects; correction to Gibbs Free energies (G corr.) at the same level of theory; Grimme's D3 dispersion correction; BP86 Electronic energies (E) computed with BS2 and including solvent effects; and final Gibbs Free energies obtained as the sum of the three previous columns. All values are given in Hartrees.

Configuration	E_{solv}(BS1/PCM)	G corr (BS1/PCM)	D3 corr (0)	E_{solv}(BS2/PCM)	G_{solv}(BS2)
Intermediate: I1					
Closed-shell singlet	-2.708.8842	0.3725	-0.1388	-2709.2824	-2709.0487
Open-shell singlet	-2708.8842	0.3725	-0.1388	-2709.2824	-2709.0487
Triplet	-2708.4965	0.3694	-0.1377	-2709.2670	-2709.0353
Intermediate: I2					
Open-shell singlet	-3248.3316	-	-	-3248.8685	-
Triplet	-3248.3343	-	-	-3.248.8746	-

4. 3D views of Mo_3S_4 species along the reaction pathway – Figure 3

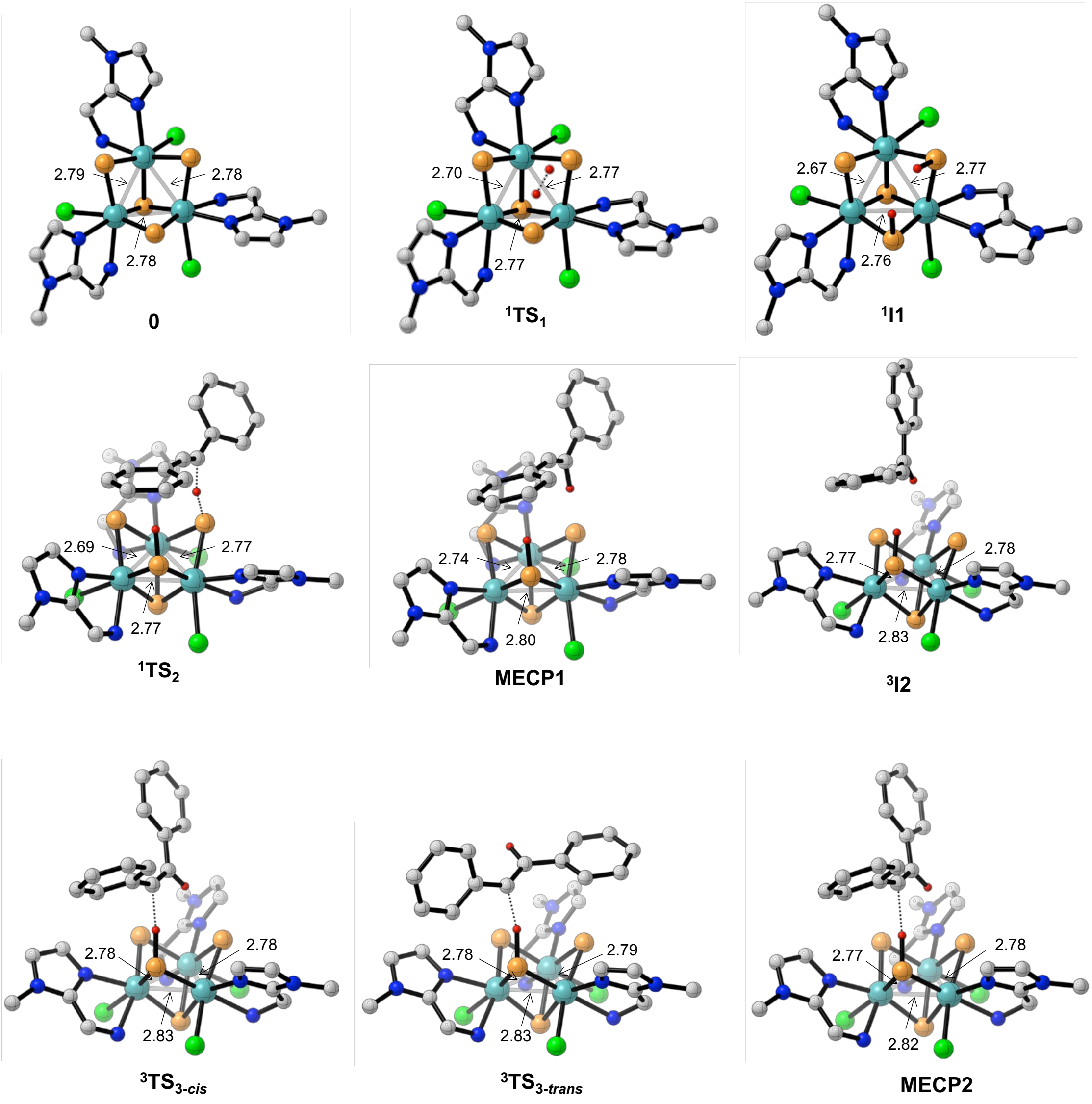

Figure S12. Optimized structures of intermediates and transition states of Gibbs energy profile of Figure 3. Selected distances are given in Å. H-atoms (except the vinilyc ones) have been omitted for clarity. Color code: Mo (cyan), S (orange), N (blue), Cl (green), H (red), O (red) and C (gray).

5. Spin densities

Table S4. Mulliken spin density of all the species in the bishydrosulfido-mediated mechanism – Figure 3.

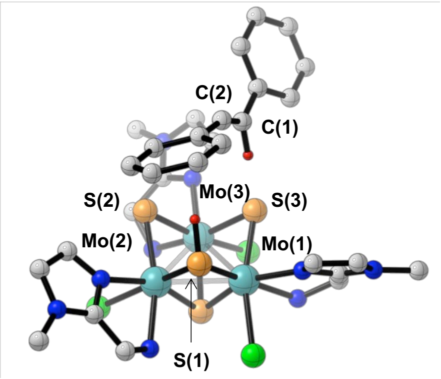

Species	Mo$_3$S$_4$ unit							DPA	
	Mo(1)	Mo(2)	Mo(3)	(μ_3-S)	S(1)	S(2)	S(3)	C (1)	C (2)
**1$^+$**	0	0	0	0	0	0	0	0	0
**TS$_1$**	0	0	0	0	0	0	0	0	0
**I1**	0	0	0	0	0	0	0	0	0
**^{1}TS$_2$**	0	0	0	0	0	0	0	0	0
**MECP1**	0.64	0.45	0.07	-0.01	0.02	-0.02	0.02	-0.10	0.61
**^{3}I2**	0.43	0.54	-0.05	-0.04	0.02	0.02	-0.01	-0.10	0.65
**^{3}TS$_{3\text{-}cis}$**	0.53	0.46	0.00	-0.01	0.02	0.01	0.09	-0.07	0.60
**MECP2**	0.50	0.48	0.00	-0.01	0.02	0.01	0.03	-0.09	0.65

Table S5. Mulliken charges of all the species in the bishydrosulfido-mediated mechanism – Figure 3.

Species	Mo$_3$S$_4$ unit						
	Mo(1)	Mo(2)	Mo(3)	(μ_3-S)	S(1)	S(2)	S(3)
**1$^+$**	0.01	0.01	0.01	0.23	-0.08	-0.08	-0.08
**TS$_1$**	-0.01	-0.03	-0.02	0.19	-0.01	-0.10	0.00
**I1**	0.01	-0.05	-0.04	0.18	-0.04	-0.15	-0.04
**^{1}TS$_2$**	0.08	-0.06	0.02	0.19	-0.15	-0.20	-0.18
**MECP1**	0.04	-0.02	0.01	0.19	-0.15	-0.13	-0.17
**^{3}I2**	0.01	0.02	-0.01	0.19	-0.15	-0.13	-0.12
**^{3}TS$_{3\text{-}cis}$**	0.05	0.04	0.04	0.19	-0.14	-0.13	-0.14
**MECP2**	0.04	0.03	-0.05	0.20	-0.13	-0.13	-0.13

6. Absolute energies of the computed species

Table S6. BP86 Electronic energies (E) computed with BS1 and including solvent effects; correction to Gibbs Free energies (G corr.) at the same level of theory; Grimme's D3 dispersion correction; BP86 Electronic energies (E) computed with BS2 and including solvent effects; and final Gibbs Free energies obtained as the sum of the three previous columns. All values are given in Hartrees.

Species	E_{solv} (BS1/PCM)	G corr (BS1/PCM)	D3 corr (0)	E_{solv}(BS2/PCM)	G_{solv}(BS2)
Reagents and products					
**dpa**	-539.4537	0.1382	-0.0166	-539.6031	-539.4815
**(Z)-stilbene**	-540.6940	0.1619	-0.0231	-540.8421	-540.7033
**(E)-stilbene**	-540.7031	0.1606	-0.0204	-540.8513	-540.7111
**H_2**	-1.1766	-0.0039	-0.0001	-1.1780	-1.1819
Pathway A : Dithiolene-mediated mechanism					
**1^+**	-2707.7245	0.3570	-0.1327	-2708.1210	-2707.8968
**TS_I**	-3247.1555	0.5203	-0.1824	-3247.6942	-3247.3563
**I**	-3247.1803	0.5264	-0.1854	-3247.7173	-3247.3762
**TS_{II}**	-3248.3060	0.5380	-0.1914	-3248.8441	-3248.4975
**II**	-3.248.3669	0.5449	-0.1911	-3248.8697	-3248.5159
**$TS_{II\text{-}cis}$**	-3248.3196	0.5439	-0.1936	-3248.8581	-3248.5079
**TS_{III}**	-3248.3238	0.5470	-0.1963	-3248.8618	-3248.5111
**III**	-3248.3608	0.5433	-0.1951	-3248.8983	-3248.5501
**$TS_{III\text{-}trans}$**	-3248.3439	0.5422	-0.1954	-3248.8759	-3248.5291
Pathway B : Bis(hydrosulfido)-mediated mechanism					
**1^+**	-2707.7245	0.3570	-0.1327	-2708.1210	-2707.8968
**TS_1**	-2708.8762	0.3681	-0.1375	-2709.2751	-2709.0445
**^{1}I1**	-2708.8842	0.3725	-0.1388	-2709.2824	-2709.0487
**$^1TS_2$**	-3248.3204	0.5291	-0.1810	-3248.8601	-3248.5121
**$^3TS_2$**	-3248.3122	0.5304	-0.1774	-3248.8520	-3248.4990
**MECP1**	-3248.3272	-	-	-3248.8675	-
**^{1}I2**	-3248.3317	0.5315	-0.1739	-3248.8709	-3248.5133
**^{3}I2**	-3248.3343	0.5286	-0.1708	-3248.8746	-3248.5168
**$TS_{3\text{-}cis}$**	-3248.3333	0.5328	-0.1761	-3248.8727	-3248.5161
**$TS_{3\text{-}trans}$**	-3248.3257	0.5348	-0.1828	-3248.8637	-3248.5117
**MECP2**	-3248.3336	-	-	-3248.8734	-

Table S7. Absolute energies of selected intermediates and transition states for the semihydrogenation of dpa catalyzed by $[Mo_3S_4Cl_3(dmen)_3]^+$ cluster.

Species	E_{solv} (BS1/PCM)	G corr (BS1/PCM)	D3 corr (0)	E_{solv}(BS2/PCM)	G_{solv}(BS2)
Pathway B : Bis(hydrosulfido)-mediated mechanism					
**Mo_3S_4-dmen**	-2434.5020	0.4358	-0.1576	-2434.8227	-2434.5445
**$^1TS2_{dmen}$**	-2975.0885	0.6116	-0.2071	-2975.5523	-2975.1479
**$^3I2_{dmen}$**	-2975.1021	0.6075	-0.1933	-2975.5676	-2975.1534

7. Cartesian coordinates of the optimized species

Stationary point: diphenylacetylene (dpa)

C	-4.7547	1.3973	-0.8662
C	-3.6460	1.2042	0.0014
C	-3.2594	2.2628	0.8669
C	-3.9629	3.4738	0.8597
C	-5.0587	3.6545	-0.0035
C	-5.4501	2.6129	-0.8640
H	-5.0571	0.5857	-1.5348
H	-2.4065	2.1198	1.5373
H	-3.6547	4.2815	1.5314
H	-5.6053	4.6028	-0.0055
H	-6.3025	2.7488	-1.5375
C	-2.9335	-0.0306	0.0027
C	-2.3187	-1.0974	0.0026
C	-1.6062	-2.3322	0.0042
C	-1.9956	-3.3927	-0.8577
C	-0.4948	-2.5235	0.8687
C	-1.2922	-4.6036	-0.8501
H	-2.8507	-3.2511	-1.5256
C	0.2005	-3.7392	0.8670
H	-0.1901	-1.7104	1.5345
C	-0.1937	-4.7825	0.0100
H	-1.6026	-5.4129	-1.5190
H	1.0551	-3.8736	1.5380
H	0.3529	-5.7309	0.0124

Stationary point: hydrogen (H_2)

H	0.7135	1.2738	0.0000
H	-0.0374	1.2738	0.0000

Stationary point: (*Z*)-stilbene

C	-0.7999	0.6596	0.3952
H	-0.1773	-0.2423	0.3122
C	-0.1369	1.7833	0.7915
H	0.9520	1.6698	0.8888
C	-0.6152	3.1333	1.1466
C	0.2785	4.2247	1.0043
C	-1.8921	3.3909	1.7065
C	-0.1000	5.5263	1.3641
H	1.2806	4.0418	0.5993
C	-2.2663	4.6897	2.0757
H	-2.5861	2.5608	1.8656
C	-1.3770	5.7658	1.8993
H	0.6062	6.3531	1.2341
H	-3.2555	4.8630	2.5130
H	-1.6736	6.7794	2.1883
C	-2.2111	0.4298	0.0285
C	-2.7558	-0.8660	0.2132
C	-3.0304	1.4124	-0.5825
C	-4.0770	-1.1588	-0.1547
H	-2.1291	-1.6471	0.6590
C	-4.3470	1.1164	-0.9604
H	-2.6221	2.4087	-0.7749
C	-4.8810	-0.1669	-0.7416
H	-4.4764	-2.1652	0.0094
H	-4.9585	1.8896	-1.4378
H	-5.9104	-0.3935	-1.0375

Stationary point: (*E*)-stilbene

c	-1.5779	1.5852	0.7381
H	-1.1412	0.5773	0.7402
C	-0.7434	2.6614	0.7381
H	-1.1800	3.6692	0.7402
C	0.7239	2.6399	0.7378
C	1.4204	3.8757	0.7524
C	1.4939	1.4471	0.7232
C	2.8215	3.9227	0.7537
H	0.8443	4.8084	0.7633
C	2.8929	1.4950	0.7247
H	0.9942	0.4729	0.7095
C	3.5663	2.7315	0.7400
H	3.3326	4.8912	0.7654
H	3.4648	0.5610	0.7130
H	4.6606	2.7633	0.7408

C	-3.0452	1.6066	0.7378
C	-3.7417	0.3708	0.7524
C	-3.8152	2.7994	0.7232
C	-5.1428	0.3238	0.7537
H	-3.1656	-0.5618	0.7633
C	-5.2141	2.7516	0.7247
H	-3.3155	3.7736	0.7095
C	-5.8876	1.5150	0.7400
H	-5.6538	-0.6447	0.7654
H	-5.7861	3.6855	0.7130
H	-6.9819	1.4833	0.7408

Stationary point: 1^{+} cluster

Mo	0.4357	1.5279	0.8999
Mo	1.2480	-1.1358	0.7824
S	-0.3403	-2.0610	2.2218
S	2.0632	0.5012	2.2233
S	-1.3594	1.3019	2.3649
S	0.0142	0.0198	-0.8882
Cl	1.0222	-3.1359	-0.8102
Cl	2.2391	2.4637	-0.6672
Mo	-1.4619	-0.5128	0.8954
Cl	-3.2058	0.7058	-0.5403
N	-3.2920	-1.2594	1.8836
C	-3.8071	-2.4023	1.3953
C	0.0231	4.4910	1.5148
N	0.7918	3.4606	1.9123
C	4.0607	-2.2700	1.1364
N	2.8230	-2.4251	1.6404
C	2.8866	-3.4541	2.5654
H	2.0094	-3.7895	3.1126
N	3.0933	-0.8054	-0.5237
H	3.1160	0.1943	-0.7963
H	2.9531	-1.3612	-1.3776
C	4.3735	-1.1970	0.1393
H	4.7782	-0.3062	0.6526
H	5.1246	-1.5236	-0.5993
N	4.9143	-3.1650	1.7056
C	6.3529	-3.2863	1.4412
H	6.7162	-4.2077	1.9139
H	6.5324	-3.3472	0.3577
H	6.8953	-2.4244	1.8589
C	4.1837	-3.9189	2.6166
H	4.6478	-4.7085	3.2018
N	-0.8061	3.0644	-0.2498

H	-1.6878	2.6074	-0.5469
H	-0.2745	3.3015	-1.0979
C	-1.0968	4.2997	0.5386
H	-2.0485	4.1423	1.0777
H	-1.2294	5.1745	-0.1194
N	0.3932	5.6291	2.1642
C	-0.2316	6.9512	2.0391
H	-0.4339	7.1703	0.9808
H	-1.1717	6.9894	2.6107
H	0.4647	7.7043	2.4301
C	1.6830	3.9552	2.8497
H	2.4313	3.3228	3.3203
C	1.4407	5.3020	3.0178
H	1.9136	6.0499	3.6488
N	-2.1944	-2.2510	-0.3963
H	-1.3665	-2.7592	-0.7582
H	-2.6826	-1.8327	-1.1993
C	-3.1037	-3.1867	0.3310
H	-3.8107	-3.6786	-0.3577
H	-2.4815	-3.9767	0.7888
N	-4.9646	-2.7096	2.0429
C	-5.8012	-3.8947	1.8179
H	-5.9807	-4.0277	0.7410
H	-5.3137	-4.7934	2.2258
H	-6.7639	-3.7422	2.3225
C	-5.1896	-1.7177	2.9905
C	-4.1481	-0.8207	2.8800
H	-3.9650	0.0977	3.4320
H	-6.0616	-1.7384	3.6387

Stationary point: TS_I

Mo	0.6031	1.5892	0.8336
Mo	1.3329	-1.0353	0.8624
S	2.1965	0.6322	2.2365
S	0.0680	0.0083	-0.8648
Cl	0.9837	-3.1713	-0.5332
Cl	2.3602	2.4027	-0.8371
Mo	-1.3644	-0.3722	0.9911
Cl	-3.1375	0.8369	-0.4452
N	-3.1955	-1.0110	2.0627
C	-3.7310	-2.1855	1.6842
C	0.2500	4.5888	1.3020
N	1.0340	3.5754	1.7131
C	4.1325	-2.2187	1.1825
N	2.9150	-2.3123	1.7465

C	2.9866	-3.3097	2.7041
H	2.1268	-3.5864	3.3092
N	3.1294	-0.8389	-0.5233
H	3.1554	0.1396	-0.8665
H	2.9525	-1.4508	-1.3304
C	4.4261	-1.2021	0.1230
H	4.8464	-0.2870	0.5781
H	5.1560	-1.5711	-0.6168
N	4.9825	-3.1214	1.7463
C	6.4027	-3.3022	1.4245
H	6.7545	-4.2214	1.9101
H	6.5329	-3.4022	0.3368
H	6.9932	-2.4485	1.7909
C	4.2694	-3.8161	2.7164
H	4.7343	-4.5977	3.3116
N	-0.6966	3.0778	-0.3254
H	-1.5968	2.6117	-0.5501
H	-0.2122	3.2682	-1.2122
C	-0.9336	4.3526	0.4147
H	-1.8468	4.2279	1.0242
H	-1.1093	5.1957	-0.2741
N	0.6831	5.7643	1.8371
C	0.0722	7.0862	1.6542
H	-0.1458	7.2542	0.5894
H	-0.8569	7.1662	2.2391
H	0.7846	7.8486	1.9949
C	2.0022	4.1208	2.5398
H	2.7784	3.5094	2.9929
C	1.7901	5.4807	2.6286
H	2.3186	6.2633	3.1666
N	-2.1657	-2.1833	-0.1489
H	-1.3502	-2.7228	-0.4938
H	-2.6743	-1.8201	-0.9659
C	-3.0585	-3.0564	0.6679
H	-3.7866	-3.5959	0.0389
H	-2.4285	-3.8133	1.1689
N	-4.8820	-2.4207	2.3734
C	-5.7303	-3.6142	2.2780
H	-5.8112	-3.9296	1.2281
H	-5.3096	-4.4361	2.8777
H	-6.7318	-3.3639	2.6517
C	-5.0810	-1.3465	3.2335
C	-4.0318	-0.4759	3.0281
H	-3.8316	0.4874	3.4895
H	-5.9426	-1.3002	3.8945
S	-1.1302	1.4286	2.4385

S	-0.2405	-1.7466	2.4776
C	0.0635	-0.5636	4.6242
C	-0.4493	0.6007	4.6502
C	0.8341	-1.5464	5.3605
C	0.3573	-2.8657	5.5683
C	2.0759	-1.1668	5.9336
C	1.0945	-3.7697	6.3464
H	-0.5975	-3.1679	5.1275
C	2.8086	-2.0811	6.7033
H	2.4508	-0.1522	5.7685
C	2.3227	-3.3840	6.9129
H	0.7068	-4.7800	6.5122
H	3.7627	-1.7727	7.1431
H	2.8971	-4.0946	7.5154
C	-0.9732	1.6593	5.4893
C	-1.9132	1.3318	6.5019
C	-0.5494	3.0059	5.3578
C	-2.4004	2.3246	7.3636
H	-2.2487	0.2953	6.6043
C	-1.0363	3.9884	6.2308
H	0.1681	3.2715	4.5753
C	-1.9650	3.6549	7.2334
H	-3.1227	2.0557	8.1412
H	-0.6894	5.0217	6.1268
H	-2.3475	4.4275	7.9077

Stationary point: I

Mo	0.6030	1.5059	0.6715
Mo	1.3294	-1.0585	0.6840
S	2.1985	0.5776	2.1150
S	0.0540	-0.0403	-1.0668
Cl	0.9337	-3.2510	-0.5823
Cl	2.3313	2.3447	-0.9712
Mo	-1.3911	-0.4279	0.7713
Cl	-3.1462	0.8508	-0.5806
N	-3.2392	-1.1123	1.7927
C	-3.7429	-2.2922	1.3894
C	0.1975	4.4904	1.2290
N	1.0115	3.4816	1.5927
C	4.1407	-2.2065	0.9919
N	2.9309	-2.3148	1.5669
C	3.0277	-3.2986	2.5362
H	2.1760	-3.5894	3.1459
N	3.0891	-0.8627	-0.7156
H	3.0952	0.1164	-1.0616

H	2.9205	-1.4788	-1.5212
C	4.4016	-1.1940	-0.0794
H	4.8045	-0.2655	0.3642
H	5.1296	-1.5529	-0.8258
N	5.0129	-3.0849	1.5610
C	6.4347	-3.2401	1.2332
H	6.8031	-4.1585	1.7080
H	6.5624	-3.3269	0.1441
H	7.0127	-2.3805	1.6059
C	4.3206	-3.7797	2.5459
H	4.8042	-4.5448	3.1476
N	-0.7496	3.0046	-0.4174
H	-1.6471	2.5320	-0.6472
H	-0.2791	3.2264	-1.3041
C	-0.9967	4.2572	0.3559
H	-1.9003	4.1065	0.9736
H	-1.1938	5.1144	-0.3097
N	0.6232	5.6604	1.7810
C	-0.0183	6.9738	1.6469
H	-0.2315	7.1815	0.5880
H	-0.9538	7.0064	2.2260
H	0.6718	7.7380	2.0268
C	1.9950	4.0271	2.4019
H	2.7956	3.4191	2.8156
C	1.7599	5.3796	2.5310
H	2.2894	6.1592	3.0725
N	-2.1411	-2.2199	-0.4073
H	-1.3080	-2.7415	-0.7402
H	-2.6416	-1.8581	-1.2301
C	-3.0299	-3.1297	0.3738
H	-3.7311	-3.6749	-0.2804
H	-2.3930	-3.8808	0.8749
N	-4.9019	-2.5603	2.0522
C	-5.7230	-3.7698	1.9262
H	-5.7708	-4.0780	0.8721
H	-5.3006	-4.5880	2.5299
H	-6.7385	-3.5429	2.2764
C	-5.1406	-1.5010	2.9211
C	-4.1063	-0.6062	2.7473
H	-3.9383	0.3568	3.2225
H	-6.0158	-1.4805	3.5652
S	-1.1799	1.2925	2.3932
S	-0.2924	-1.7961	2.3706
C	-0.1766	-0.8104	3.9493
C	-0.6161	0.4700	3.9761
C	0.4477	-1.5613	5.0640

C	0.0624	-2.8924	5.3560
C	1.4779	-0.9677	5.8339
C	0.6769	-3.5999	6.4018
H	-0.7335	-3.3660	4.7718
C	2.0927	-1.6808	6.8723
H	1.7975	0.0525	5.6018
C	1.6938	-2.9982	7.1620
H	0.3564	-4.6231	6.6236
H	2.8903	-1.2078	7.4544
H	2.1745	-3.5525	7.9744
C	-0.7653	1.3286	5.1718
C	-1.3935	0.8283	6.3395
C	-0.3272	2.6749	5.1621
C	-1.5596	1.6463	7.4657
H	-1.7568	-0.2038	6.3530
C	-0.4891	3.4869	6.2956
H	0.1593	3.0767	4.2673
C	-1.1060	2.9775	7.4507
H	-2.0517	1.2436	8.3571
H	-0.1310	4.5214	6.2743
H	-1.2374	3.6139	8.3316

Stationary point: TS_{II}

Mo	0.6453	1.6251	0.4161
Mo	1.1803	-0.9357	0.8887
S	2.3098	0.8624	1.9345
S	0.0434	-0.1296	-1.0733
Cl	0.5547	-3.2672	-0.0345
Cl	2.5329	2.0164	-1.2743
Mo	-1.5368	-0.0327	0.6782
Cl	-3.0850	1.1927	-0.9311
N	-3.4895	-0.3630	1.6812
C	-4.1119	-1.5217	1.3994
C	0.4551	4.6824	0.4622
N	1.1690	3.6951	1.0332
C	3.8320	-2.3388	1.4548
N	2.6324	-2.1618	2.0359
C	2.6357	-2.9055	3.2045
H	1.7731	-2.9442	3.8657
N	2.9202	-1.2524	-0.4972
H	3.0427	-0.3681	-1.0252
H	2.6499	-1.9870	-1.1648
C	4.1953	-1.6236	0.1913
H	4.7399	-0.6906	0.4234
H	4.8405	-2.2312	-0.4648

N	4.6037	-3.1705	2.2085
C	5.9896	-3.5661	1.9336
H	6.2194	-4.4656	2.5193
H	6.1064	-3.7989	0.8654
H	6.6845	-2.7591	2.2132
C	3.8582	-3.5331	3.3239
H	4.2582	-4.2014	4.0820
N	-0.4647	3.0115	-1.0144
H	-1.3817	2.5810	-1.2489
H	0.1015	3.0436	-1.8724
C	-0.6677	4.3913	-0.4842
H	-1.6364	4.4166	0.0467
H	-0.7224	5.1338	-1.2987
N	0.8911	5.8959	0.9012
C	0.3403	7.2061	0.5356
H	0.2243	7.2732	-0.5561
H	-0.6352	7.3635	1.0211
H	1.0408	7.9834	0.8672
C	2.0959	4.2999	1.8670
H	2.8170	3.7214	2.4387
C	1.9291	5.6667	1.7972
H	2.4498	6.4865	2.2852
N	-2.4190	-1.9018	-0.2774
H	-1.6358	-2.5555	-0.4623
H	-2.8241	-1.6230	-1.1808
C	-3.4561	-2.5739	0.5606
H	-4.1849	-3.1241	-0.0580
H	-2.9464	-3.3126	1.2053
N	-5.3325	-1.5591	2.0026
C	-6.2942	-2.6670	1.9593
H	-6.4611	-2.9806	0.9181
H	-5.9256	-3.5208	2.5483
H	-7.2451	-2.3188	2.3823
C	-5.4892	-0.3674	2.7022
C	-4.3425	0.3685	2.4912
H	-4.0810	1.3616	2.8474
H	-6.3922	-0.1558	3.2690
S	-1.1216	1.8273	2.1338
S	-0.6686	-1.3570	2.4358
C	-0.5046	-0.3254	3.9131
C	-0.6047	1.0814	3.8210
C	-0.0804	-1.0296	5.1263
C	-0.4054	-2.4012	5.3370
C	0.6878	-0.3696	6.1282
C	0.0203	-3.0749	6.4886
H	-1.0173	-2.9289	4.5987

C	1.1228	-1.0565	7.2683
H	0.9536	0.6834	6.0029
C	0.7918	-2.4102	7.4591
H	-0.2577	-4.1241	6.6316
H	1.7228	-0.5281	8.0163
H	1.1239	-2.9389	8.3578
C	-1.1619	1.9475	4.9136
C	-2.2381	1.4905	5.7090
C	-0.6666	3.2548	5.1232
C	-2.7965	2.3208	6.6935
H	-2.6354	0.4837	5.5478
C	-1.2274	4.0807	6.1086
H	0.1699	3.6185	4.5158
C	-2.2947	3.6173	6.8981
H	-3.6281	1.9505	7.3019
H	-0.8251	5.0871	6.2641
H	-2.7301	4.2615	7.6686
H	1.5917	1.3892	3.4054
H	0.7895	1.4527	3.8652

Stationary point: II

Mo	0.7976	1.6222	0.4784
Mo	1.2265	-0.9536	0.9077
S	2.6267	0.8186	1.8815
S	0.0440	-0.0994	-0.9824
Cl	0.4149	-3.2644	0.1185
Cl	2.6726	1.9177	-1.2569
Mo	-1.4681	0.0573	0.8334
Cl	-2.9989	1.3306	-0.7564
N	-3.4209	-0.1743	1.8675
C	-4.0789	-1.3254	1.6347
C	0.7449	4.6853	0.4653
N	1.4340	3.6821	1.0385
C	3.8561	-2.4937	1.2567
N	2.7237	-2.2529	1.9407
C	2.8033	-2.9849	3.1146
H	2.0132	-2.9672	3.8609
N	2.8458	-1.3534	-0.6095
H	2.9794	-0.4736	-1.1430
H	2.4769	-2.0632	-1.2559
C	4.1498	-1.8068	-0.0381
H	4.7750	-0.9130	0.1381
H	4.6944	-2.4573	-0.7429
N	4.6557	-3.3531	1.9471
C	5.9803	-3.8306	1.5348

H	6.2955	-4.6221	2.2269
H	5.9316	-4.2457	0.5171
H	6.7129	-3.0095	1.5645
C	4.0006	-3.6695	3.1306
H	4.4372	-4.3483	3.8586
N	-0.3042	3.0216	-0.9331
H	-1.2473	2.6324	-1.1340
H	0.2388	3.0077	-1.8067
C	-0.4242	4.4219	-0.4316
H	-1.3693	4.5036	0.1352
H	-0.4776	5.1453	-1.2633
N	1.2425	5.8892	0.8641
C	0.7383	7.2122	0.4782
H	0.6601	7.2812	-0.6171
H	-0.2477	7.3957	0.9320
H	1.4469	7.9715	0.8330
C	2.4081	4.2658	1.8325
H	3.1187	3.6696	2.3990
C	2.2960	5.6367	1.7353
H	2.8640	6.4447	2.1888
N	-2.4265	-1.8075	-0.0434
H	-1.6664	-2.4867	-0.2424
H	-2.8622	-1.5391	-0.9358
C	-3.4426	-2.4311	0.8535
H	-4.1844	-3.0175	0.2857
H	-2.9142	-3.1286	1.5288
N	-5.3237	-1.2767	2.1848
C	-6.3262	-2.3482	2.1803
H	-6.4180	-2.7733	1.1701
H	-6.0473	-3.1417	2.8905
H	-7.2936	-1.9205	2.4738
C	-5.4614	-0.0344	2.7950
C	-4.2792	0.6427	2.5845
H	-3.9933	1.6458	2.8874
H	-6.3759	0.2462	3.3111
S	-0.9086	1.9661	2.1708
S	-0.5217	-1.2076	2.6072
C	-0.1646	-0.1575	3.9881
C	-0.4065	1.3159	3.9414
C	0.3714	-0.7687	5.1869
C	0.2702	-2.1763	5.4353
C	1.0130	0.0203	6.1963
C	0.8010	-2.7519	6.5931
H	-0.2697	-2.8062	4.7206
C	1.5596	-0.5710	7.3401
H	1.0889	1.1044	6.0764

C	1.4616	-1.9587	7.5518
H	0.6885	-3.8289	6.7562
H	2.0569	0.0622	8.0823
H	1.8741	-2.4125	8.4579
C	-1.4689	1.8642	4.8844
C	-2.4848	1.0508	5.4268
C	-1.4329	3.2327	5.2356
C	-3.4449	1.5945	6.2967
H	-2.5159	-0.0138	5.1748
C	-2.3924	3.7753	6.1033
H	-0.6415	3.8734	4.8290
C	-3.4043	2.9567	6.6366
H	-4.2235	0.9476	6.7140
H	-2.3440	4.8359	6.3712
H	-4.1491	3.3766	7.3203
H	2.2259	0.8639	3.1920
H	0.5388	1.8610	4.1538

Stationary point: $TS_{II\text{-}cis}$

Mo	0.7775	1.6983	0.4727
Mo	1.0697	-1.0820	0.9575
S	2.3946	0.7192	1.9739
S	0.0799	-0.0833	-0.9591
Cl	0.3502	-3.3031	-0.0563
Cl	2.7143	1.9420	-1.1898
Mo	-1.3759	0.0466	0.9062
Cl	-2.8934	1.3242	-0.7101
N	-3.3613	-0.1103	1.9246
C	-4.0655	-1.2285	1.6712
C	0.8017	4.7536	0.4665
N	1.4435	3.7275	1.0530
C	3.6960	-2.6090	1.3849
N	2.4819	-2.5052	1.9608
C	2.4128	-3.5059	2.9159
H	1.5197	-3.6730	3.5070
N	2.8472	-1.2948	-0.4381
H	2.9966	-0.3909	-0.9255
H	2.5850	-1.9935	-1.1460
C	4.1014	-1.7207	0.2527
H	4.6096	-0.8138	0.6256
H	4.7938	-2.2267	-0.4410
N	4.4058	-3.6254	1.9490
C	5.7805	-4.0211	1.6232
H	5.9715	-5.0087	2.0623
H	5.9040	-4.0875	0.5324

H	6.4993	-3.2944	2.0320
C	3.6035	-4.1994	2.9274
H	3.9480	-5.0387	3.5259
N	-0.2611	3.1131	-0.9408
H	-1.2073	2.7265	-1.1345
H	0.2743	3.0926	-1.8195
C	-0.3665	4.5153	-0.4389
H	-1.3130	4.6064	0.1241
H	-0.4056	5.2400	-1.2696
N	1.3436	5.9381	0.8650
C	0.8976	7.2804	0.4736
H	0.7337	7.3186	-0.6131
H	-0.0334	7.5461	0.9976
H	1.6830	7.9998	0.7391
C	2.4302	4.2740	1.8576
H	3.1087	3.6527	2.4366
C	2.3741	5.6479	1.7521
H	2.9677	6.4348	2.2100
N	-2.4138	-1.7692	0.0136
H	-1.6833	-2.4795	-0.1986
H	-2.8298	-1.4658	-0.8765
C	-3.4665	-2.3572	0.8940
H	-4.2255	-2.9050	0.3108
H	-2.9798	-3.0837	1.5689
N	-5.3266	-1.1179	2.1725
C	-6.3800	-2.1380	2.1257
H	-6.4691	-2.5402	1.1056
H	-6.1577	-2.9567	2.8272
H	-7.3317	-1.6679	2.4054
C	-5.4274	0.1336	2.7707
C	-4.2070	0.7516	2.6027
H	-3.8840	1.7395	2.9192
H	-6.3448	0.4592	3.2541
S	-0.8824	2.0063	2.1746
S	-0.5651	-1.3020	2.8010
C	-0.0267	-0.0867	4.0578
C	-0.4473	1.3330	3.9931
C	0.7481	-0.5613	5.1876
C	0.8166	-1.9540	5.5137
C	1.4032	0.3373	6.0957
C	1.4941	-2.4129	6.6466
H	0.2824	-2.6693	4.8824
C	2.0993	-0.1344	7.2122
H	1.3672	1.4170	5.9237
C	2.1574	-1.5118	7.5008
H	1.5031	-3.4858	6.8670

H	2.5955	0.5844	7.8729
H	2.6976	-1.8735	8.3807
C	-1.6677	1.7108	4.8261
C	-2.4781	0.7397	5.4483
C	-1.9618	3.0777	5.0368
C	-3.5631	1.1251	6.2538
H	-2.2490	-0.3213	5.3091
C	-3.0457	3.4613	5.8401
H	-1.3303	3.8448	4.5735
C	-3.8532	2.4846	6.4511
H	-4.1793	0.3567	6.7323
H	-3.2525	4.5248	5.9991
H	-4.6940	2.7835	7.0852
H	1.8379	0.7612	3.2296
H	0.3773	2.0098	4.2911

Stationary point: TS_{III}

Mo	0.8521	1.6096	0.634
Mo	1.1807	-0.9976	1.135
S	2.6428	0.7447	2.0564
S	0.0317	-0.1481	-0.7531
Cl	0.2703	-3.2843	0.3892
Cl	2.7312	1.8047	-1.1217
Mo	-1.4633	0.1808	1.0522
Cl	-2.8744	1.3332	-0.7466
N	-3.4793	0.1819	1.9665
C	-4.2034	-0.9458	1.8212
C	0.9395	4.6687	0.5326
N	1.5806	3.6536	1.1392
C	3.8033	-2.5966	1.3709
N	2.6675	-2.4084	2.068
C	2.6764	-3.3393	3.0932
H	1.8627	-3.4168	3.805
N	2.7756	-1.3884	-0.4207
H	2.89	-0.5177	-0.9749
H	2.4202	-2.1231	-1.0474
C	4.0957	-1.7933	0.1469
H	4.6496	-0.8726	0.4029
H	4.6991	-2.3544	-0.5862
N	4.5415	-3.6002	1.9206
C	5.8564	-4.0702	1.4707
H	6.0648	-5.0335	1.9541
H	5.8518	-4.2137	0.3803
H	6.6402	-3.3472	1.7442
C	3.8391	-4.0759	3.0207

H	4.225	-4.8848	3.6354
N	-0.1675	3.0157	-0.8307
H	-1.118	2.6562	-1.0486
H	0.3944	2.9695	-1.6913
C	-0.2465	4.4252	-0.3484
H	-1.1816	4.5339	0.2307
H	-0.2943	5.1386	-1.1888
N	1.4992	5.8599	0.8868
C	1.0579	7.1947	0.4662
H	0.8717	7.2037	-0.6175
H	0.1404	7.4848	1.0011
H	1.8554	7.9137	0.6944
C	2.5857	4.2147	1.9093
H	3.2685	3.6035	2.4938
C	2.5414	5.5855	1.7649
H	3.1506	6.3794	2.1888
N	-2.49	-1.7394	0.3543
H	-1.7645	-2.4772	0.2422
H	-2.8797	-1.5363	-0.5758
C	-3.5764	-2.1878	1.2718
H	-4.3093	-2.8324	0.7584
H	-3.1131	-2.7847	2.0783
N	-5.5032	-0.7165	2.1542
C	-6.5943	-1.6969	2.1656
H	-6.5418	-2.3268	1.2658
H	-6.5361	-2.3318	3.063
H	-7.5495	-1.1557	2.1626
C	-5.6077	0.6199	2.5278
C	-4.3505	1.1664	2.3993
H	-4.0129	2.1786	2.6023
H	-6.5523	1.0441	2.8572
S	-0.8593	2.0908	2.2393
S	-0.5524	-1.1131	2.9103
C	-0.0844	-0.0413	4.2928
C	-0.6637	1.2351	4.4836
C	0.9616	-0.5324	5.2042
C	0.9902	-1.8979	5.597
C	1.9107	0.3451	5.8046
C	1.9225	-2.3645	6.5338
H	0.2359	-2.5825	5.1944
C	2.8598	-0.1357	6.7167
H	1.908	1.413	5.5623
C	2.8731	-1.4913	7.0901
H	1.8996	-3.4169	6.8354
H	3.585	0.5609	7.1501
H	3.6031	-1.8577	7.8182

C	-2.0744	1.471	4.8918
C	-2.9216	0.4026	5.2704
C	-2.5342	2.7979	5.0851
C	-4.1882	0.6547	5.8189
H	-2.5672	-0.6277	5.1643
C	-3.8013	3.0461	5.629
H	-1.881	3.6356	4.8157
C	-4.6347	1.9745	5.9997
H	-4.8225	-0.1855	6.12
H	-4.135	4.0784	5.7776
H	-5.6182	2.1686	6.4394
H	2.2671	0.873	3.3705
H	0.0092	1.9598	4.9683

Stationary point: III

Mo	0.5720	1.4321	-1.3088
Mo	1.1189	-1.2157	-0.5998
S	2.1343	0.7246	0.4421
S	-0.0098	-0.5152	-2.5531
Cl	0.5292	-3.5727	-1.4170
Cl	2.5584	1.6636	-2.9206
Mo	-1.4792	-0.1790	-0.7158
Cl	-3.0303	0.6763	-2.5524
N	-3.4684	-0.3096	0.2766
C	-4.0832	-1.5076	0.2360
C	0.4536	4.4617	-1.6898
N	1.1249	3.5569	-0.9580
C	3.9097	-2.4390	-0.1859
N	2.6998	-2.4494	0.4078
C	2.7513	-3.4212	1.3943
H	1.8939	-3.6548	2.0176
N	2.8579	-1.3783	-2.0458
H	2.8864	-0.5127	-2.6201
H	2.6660	-2.1744	-2.6696
C	4.1726	-1.5673	-1.3687
H	4.5400	-0.5725	-1.0589
H	4.9245	-2.0036	-2.0478
N	4.7323	-3.3553	0.3930
C	6.1348	-3.6167	0.0512
H	6.4329	-4.5671	0.5125
H	6.2472	-3.7015	-1.0397
H	6.7820	-2.8095	0.4269
C	4.0100	-3.9812	1.4024
H	4.4541	-4.7553	2.0224
N	-0.4810	2.6088	-2.9211

H	-1.4006	2.1490	-3.0793
H	0.0708	2.5175	-3.7839
C	-0.6755	4.0512	-2.5830
H	-1.6371	4.1435	-2.0460
H	-0.7378	4.6727	-3.4918
N	0.9199	5.7160	-1.4334
C	0.4213	6.9673	-2.0158
H	1.0965	7.7809	-1.7216
H	0.4084	6.8946	-3.1135
H	-0.5928	7.1859	-1.6476
C	2.0568	4.2528	-0.2076
H	2.7453	3.7472	0.4647
C	1.9346	5.5978	-0.4907
H	2.4712	6.4681	-0.1218
N	-2.3764	-2.2201	-1.2903
H	-1.6016	-2.9058	-1.4024
H	-2.8135	-2.0963	-2.2127
C	-3.3896	-2.7145	-0.3134
H	-4.0987	-3.4186	-0.7802
H	-2.8569	-3.2592	0.4857
N	-5.3655	-1.3947	0.6807
C	-6.3401	-2.4804	0.8358
H	-6.1439	-3.0473	1.7589
H	-7.3458	-2.0428	0.8823
H	-6.2874	-3.1571	-0.0292
C	-5.5759	-0.0605	1.0131
C	-4.3979	0.6025	0.7456
H	-4.1565	1.6563	0.8477
H	-6.5309	0.2845	1.4008
S	-1.1032	1.9238	0.2497
S	-0.5377	-1.4236	1.1941
C	-0.0718	-0.8505	2.9159
C	-0.3202	0.2607	3.6643
C	0.6683	-1.9606	3.6019
C	0.0921	-3.2480	3.7227
C	1.9180	-1.7167	4.2159
C	0.7470	-4.2577	4.4459
H	-0.8810	-3.4500	3.2635
C	2.5700	-2.7288	4.9378
H	2.3781	-0.7276	4.1201
C	1.9889	-4.0026	5.0545
H	0.2804	-5.2436	4.5409
H	3.5381	-2.5211	5.4051
H	2.4979	-4.7912	5.6175
C	-1.1147	1.4987	3.5360
C	-0.5102	2.7344	3.8755

C	-2.5035	1.4727	3.2855
C	-1.2636	3.9160	3.9111
H	0.5593	2.7615	4.1120
C	-3.2622	2.6527	3.3510
H	-2.9886	0.5182	3.0628
C	-2.6462	3.8788	3.6531
H	-0.7767	4.8635	4.1649
H	-4.3428	2.6119	3.1786
H	-3.2421	4.7955	3.7099
H	1.3990	0.9022	1.5994
H	0.1611	0.1954	4.6537

Stationary point: TS $_{\text{III-}\textit{trans}}$

Mo	0.6152	1.3825	-1.9090
Mo	1.1200	-1.3197	-1.3308
S	2.0222	0.5209	-0.1187
S	0.1265	-0.4916	-3.3050
Cl	0.6071	-3.6286	-2.3550
Cl	2.6986	1.6879	-3.3772
Mo	-1.4473	-0.2962	-1.5372
Cl	-2.9037	0.6581	-3.4123
N	-3.4951	-0.5158	-0.6829
C	-4.0582	-1.7352	-0.7851
C	0.5372	4.4310	-2.1159
N	1.1507	3.4760	-1.3966
C	3.8204	-2.6600	-0.7678
N	2.5664	-2.6464	-0.2757
C	2.4839	-3.6848	0.6355
H	1.5710	-3.8980	1.1823
N	2.9846	-1.4231	-2.6321
H	3.0851	-0.5179	-3.1314
H	2.8248	-2.1610	-3.3314
C	4.2225	-1.7164	-1.8539
H	4.5901	-0.7631	-1.4334
H	5.0211	-2.1309	-2.4920
N	4.5431	-3.6610	-0.1950
C	5.9532	-3.9806	-0.4430
H	6.1534	-4.9886	-0.0569
H	6.1570	-3.9681	-1.5236
H	6.6104	-3.2576	0.0642
C	3.7070	-4.3143	0.7041
H	4.0590	-5.1505	1.3019
N	-0.3268	2.6639	-3.5141
H	-1.2353	2.2213	-3.7573
H	0.2774	2.6189	-4.3448

C	-0.5352	4.0853	-3.1021
H	-1.5284	4.1530	-2.6221
H	-0.5379	4.7602	-3.9742
N	0.9996	5.6613	-1.7558
C	0.5451	6.9555	-2.2766
H	0.4226	6.8974	-3.3676
H	-0.4108	7.2445	-1.8130
H	1.3064	7.7120	-2.0459
C	2.0362	4.1134	-0.5451
H	2.6732	3.5591	0.1397
C	1.9469	5.4738	-0.7557
H	2.4658	6.3134	-0.3007
N	-2.2731	-2.3079	-2.2657
H	-1.4790	-2.9660	-2.4094
H	-2.7088	-2.1396	-3.1822
C	-3.2775	-2.8899	-1.3282
H	-3.9270	-3.6276	-1.8285
H	-2.7313	-3.4164	-0.5258
N	-5.3771	-1.6759	-0.4516
C	-6.3204	-2.7984	-0.3917
H	-6.1904	-3.4446	-1.2718
H	-6.1622	-3.3872	0.5248
H	-7.3414	-2.3952	-0.3955
C	-5.6669	-0.3538	-0.1315
C	-4.4973	0.3563	-0.2941
H	-4.3105	1.4199	-0.1796
H	-6.6646	-0.0493	0.1741
S	-1.1744	1.7847	-0.4763
S	-0.5167	-1.4459	0.4262
C	0.1845	-0.4948	2.0858
C	-0.3930	0.3897	2.9750
C	1.1453	-1.4512	2.7501
C	0.6446	-2.6514	3.3082
C	2.4981	-1.1145	2.9695
C	1.4760	-3.4903	4.0663
H	-0.4098	-2.9103	3.1620
C	3.3289	-1.9555	3.7293
H	2.8976	-0.1785	2.5660
C	2.8222	-3.1451	4.2779
H	1.0674	-4.4075	4.5034
H	4.3726	-1.6724	3.9008
H	3.4692	-3.7946	4.8763
C	-1.4528	1.3974	2.9598
C	-2.7173	1.2031	2.3576
C	-1.2395	2.5826	3.7151
C	-3.7318	2.1615	2.5114

H	-2.9112	0.2786	1.8080
C	-2.2376	3.5575	3.8265
H	-0.2741	2.7305	4.2122
C	-3.4950	3.3451	3.2307
H	-4.7198	1.9784	2.0778
H	-2.0463	4.4704	4.3996
H	-4.2913	4.0876	3.3447
H	1.0799	0.3670	1.0552
H	0.1268	0.3732	3.9469

Stationary point: ^{1}TS1 (s = 0)

Mo	0.6379	1.7155	0.2267
Mo	1.3162	-0.9425	0.6450
S	0.1137	-0.1037	-1.2272
Cl	0.8814	-3.2480	-0.3848
Cl	2.5570	2.1733	-1.4031
Mo	-1.3192	-0.0839	0.6788
Cl	-3.0340	0.8985	-0.9161
N	-3.1476	-0.4802	1.8665
C	-3.7078	-1.6936	1.7112
C	0.3818	4.7605	0.1889
N	1.1081	3.8063	0.7967
C	4.0900	-2.1114	1.1840
N	2.8565	-2.1149	1.7205
C	2.8973	-2.9454	2.8282
H	2.0164	-3.1348	3.4361
N	3.1328	-0.9885	-0.7217
H	3.1859	-0.0730	-1.2073
H	2.9522	-1.7133	-1.4290
C	4.4153	-1.2721	-0.0122
H	4.8506	-0.3073	0.3051
H	5.1467	-1.7619	-0.6769
N	4.9195	-2.9113	1.9089
C	6.3493	-3.1355	1.6652
H	6.6640	-4.0232	2.2289
H	6.5228	-3.3143	0.5942
H	6.9374	-2.2650	1.9943
C	4.1769	-3.4421	2.9579
H	4.6207	-4.1200	3.6822
N	-0.5225	3.0206	-1.2179
H	-1.4283	2.5477	-1.4022
H	0.0047	3.0421	-2.1005
C	-0.7545	4.4110	-0.7216
H	-1.7082	4.4172	-0.1635
H	-0.8545	5.1237	-1.5572

N	0.8113	5.9946	0.5730
C	0.2447	7.2841	0.1608
H	0.0871	7.2934	-0.9275
H	-0.7117	7.4671	0.6745
H	0.9565	8.0774	0.4227
C	2.0356	4.4500	1.5982
H	2.7611	3.9008	2.1929
C	1.8562	5.8116	1.4713
H	2.3720	6.6552	1.9226
N	-2.1549	-2.0652	-0.0991
H	-1.3526	-2.6796	-0.3417
H	-2.6587	-1.8520	-0.9695
C	-3.0673	-2.7499	0.8644
H	-3.8137	-3.3717	0.3421
H	-2.4581	-3.4252	1.4922
N	-4.8469	-1.7828	2.4521
C	-5.7187	-2.9571	2.5747
H	-5.9626	-3.3503	1.5769
H	-5.2272	-3.7407	3.1716
H	-6.6472	-2.6497	3.0726
C	-5.0135	-0.5694	3.1102
C	-3.9564	0.2325	2.7357
H	-3.7299	1.2576	3.0177
H	-5.8611	-0.3920	3.7670
S	-1.0191	1.9104	1.8635
S	-0.1846	-1.2708	2.4245
H	0.3058	0.0199	3.2712
S	2.1547	0.8820	1.8744
H	1.0959	0.7349	3.0944

Stationary point: ^{1}I1 (s = 0)

Mo	0.6532	1.6960	0.2291
Mo	1.3307	-0.9551	0.6545
S	0.1428	-0.1245	-1.2311
Cl	0.8597	-3.2655	-0.3233
Cl	2.6054	2.1471	-1.3633
Mo	-1.2919	-0.0851	0.6731
Cl	-2.9896	0.9167	-0.9216
N	-3.1378	-0.4632	1.8466
C	-3.7126	-1.6686	1.6844
C	0.3620	4.7381	0.1833
N	1.1065	3.7966	0.7880
C	4.1088	-2.1319	1.1605
N	2.8835	-2.1289	1.7155
C	2.9394	-2.9544	2.8264

H	2.0674	-3.1384	3.4486
N	3.1248	-1.0037	-0.7282
H	3.1787	-0.0865	-1.2117
H	2.9299	-1.7247	-1.4358
C	4.4186	-1.2992	-0.0437
H	4.8730	-0.3392	0.2611
H	5.1302	-1.7992	-0.7223
N	4.9475	-2.9301	1.8765
C	6.3725	-3.1591	1.6107
H	6.6991	-4.0325	2.1896
H	6.5261	-3.3635	0.5411
H	6.9658	-2.2805	1.9077
C	4.2194	-3.4540	2.9391
H	4.6724	-4.1299	3.6597
N	-0.5056	2.9799	-1.2221
H	-1.4042	2.4918	-1.4094
H	0.0251	3.0107	-2.1022
C	-0.7663	4.3658	-0.7271
H	-1.7197	4.3509	-0.1687
H	-0.8807	5.0753	-1.5636
N	0.7675	5.9797	0.5703
C	0.1713	7.2592	0.1697
H	-0.0478	7.2486	-0.9077
H	-0.7563	7.4462	0.7328
H	0.8920	8.0609	0.3768
C	2.0222	4.4562	1.5902
H	2.7597	3.9193	2.1814
C	1.8163	5.8145	1.4677
H	2.3161	6.6666	1.9210
N	-2.1399	-2.0637	-0.1046
H	-1.3419	-2.6899	-0.3324
H	-2.6256	-1.8469	-0.9841
C	-3.0784	-2.7335	0.8440
H	-3.8284	-3.3408	0.3097
H	-2.4917	-3.4215	1.4793
N	-4.8607	-1.7437	2.4133
C	-5.7480	-2.9071	2.5261
H	-5.9787	-3.3024	1.5259
H	-5.2765	-3.6939	3.1351
H	-6.6819	-2.5870	3.0056
C	-5.0181	-0.5286	3.0707
C	-3.9465	0.2594	2.7075
H	-3.7085	1.2814	2.9913
H	-5.8701	-0.3404	3.7189
S	-0.9750	1.8870	1.9170
S	-0.2021	-1.3499	2.4591

H	0.0483	-0.3019	3.3557
S	2.2510	0.9056	1.8785
H	1.4682	0.9477	3.0406

Stationary point: ^{3}I1 (s = 1)

Mo	0.4048	1.5258	0.8614
Mo	1.1909	-1.2136	0.8035
S	1.9468	0.4573	2.2511
S	-0.0268	-0.0426	-0.8995
Cl	0.8547	-3.3298	-0.5661
Cl	2.2322	2.3753	-0.7172
Mo	-1.5265	-0.5303	0.8750
Cl	-3.2171	0.8270	-0.4388
N	-3.3945	-1.2795	1.8091
C	-3.8794	-2.4354	1.3231
C	0.0415	4.4967	1.5141
N	0.8152	3.4538	1.8651
C	4.0325	-2.2497	1.1621
N	2.8053	-2.4417	1.6782
C	2.9143	-3.4525	2.6189
H	2.0527	-3.8124	3.1752
N	2.9944	-0.8640	-0.5226
H	2.9848	0.1286	-0.8236
H	2.8746	-1.4474	-1.3609
C	4.2935	-1.1856	0.1414
H	4.6571	-0.2662	0.6351
H	5.0574	-1.4932	-0.5925
N	4.9240	-3.1020	1.7397
C	6.3634	-3.1748	1.4639
H	6.7673	-4.0680	1.9573
H	6.5353	-3.2576	0.3805
H	6.8754	-2.2811	1.8524
C	4.2286	-3.8672	2.6691
H	4.7265	-4.6285	3.2639
N	-0.8684	3.0837	-0.2120
H	-1.7643	2.6425	-0.4931
H	-0.3492	3.3091	-1.0716
C	-1.1143	4.3270	0.5765
H	-2.0513	4.1923	1.1464
H	-1.2509	5.2040	-0.0784
N	0.4476	5.6232	2.1632
C	-0.1696	6.9518	2.0789
H	-0.3970	7.1927	1.0304
H	-1.0950	6.9866	2.6745
H	0.5420	7.6924	2.4664

C	1.7493	3.9293	2.7709
H	2.5094	3.2817	3.2006
C	1.5270	5.2756	2.9679
H	2.0319	6.0110	3.5889
N	-2.2031	-2.2725	-0.4056
H	-1.3645	-2.7869	-0.7395
H	-2.6630	-1.8587	-1.2272
C	-3.1393	-3.2138	0.2811
H	-3.8208	-3.6990	-0.4378
H	-2.5364	-4.0104	0.7535
N	-5.0472	-2.7563	1.9464
C	-5.8571	-3.9584	1.7165
H	-6.0146	-4.1045	0.6377
H	-5.3608	-4.8447	2.1408
H	-6.8315	-3.8198	2.2024
C	-5.3121	-1.7565	2.8753
C	-4.2838	-0.8428	2.7775
H	-4.1302	0.0870	3.3193
H	-6.1989	-1.7851	3.5030
S	-0.4493	-2.1338	2.3811
H	-0.2745	-1.4095	3.5335
S	-1.4728	1.3203	2.4598
H	-0.8536	0.8245	3.5800

Stationary point: ^{1}TS2 (s = 0)

Mo	0.6457	1.5425	0.5023
Mo	1.3167	-1.1388	0.6272
S	0.1811	-0.0900	-1.1787
Cl	0.8676	-3.3225	-0.6435
Cl	2.5796	2.2023	-1.0912
Mo	-1.3175	-0.2922	0.6519
Cl	-2.9664	0.9062	-0.8748
N	-3.2154	-0.7851	1.6997
C	-3.7892	-1.9566	1.3719
C	0.3710	4.5774	0.7680
N	1.1025	3.5749	1.2854
C	4.0747	-2.3914	1.0434
N	2.8390	-2.4467	1.5711
C	2.8633	-3.4151	2.5610
H	1.9759	-3.6700	3.1346
N	3.1421	-1.0345	-0.7174
H	3.2098	-0.0703	-1.0944
H	2.9692	-1.6717	-1.5061
C	4.4153	-1.4080	-0.0323
H	4.8422	-0.4912	0.4128

H	5.1566	-1.8090	-0.7439
N	4.8907	-3.2901	1.6614
C	6.3168	-3.5062	1.3905
H	6.6287	-4.4324	1.8900
H	6.4820	-3.6129	0.3082
H	6.9147	-2.6655	1.7746
C	4.1356	-3.9423	2.6298
H	4.5673	-4.7148	3.2610
N	-0.4933	2.9898	-0.8212
H	-1.3860	2.5290	-1.0876
H	0.0635	3.1253	-1.6751
C	-0.7649	4.3036	-0.1662
H	-1.7101	4.2079	0.3982
H	-0.8980	5.1078	-0.9091
N	0.8100	5.7764	1.2425
C	0.2482	7.0967	0.9357
H	0.1170	7.2058	-0.1510
H	-0.7210	7.2315	1.4402
H	0.9502	7.8639	1.2872
C	2.0454	4.1511	2.1196
H	2.7779	3.5546	2.6581
C	1.8686	5.5190	2.1060
H	2.3934	6.3229	2.6152
N	-2.1449	-2.1573	-0.3837
H	-1.3395	-2.7580	-0.6503
H	-2.5946	-1.8373	-1.2509
C	-3.1226	-2.9242	0.4435
H	-3.8525	-3.4617	-0.1851
H	-2.5636	-3.6835	1.0197
N	-4.9777	-2.0979	2.0227
C	-5.8812	-3.2516	1.9466
H	-6.0720	-3.5110	0.8947
H	-5.4455	-4.1180	2.4678
H	-6.8321	-2.9816	2.4236
C	-5.1635	-0.9618	2.8026
C	-4.0668	-0.1533	2.5901
H	-3.8384	0.8268	3.0009
H	-6.0485	-0.8361	3.4211
S	-1.0531	1.5414	2.1206
S	-0.3107	-1.7924	2.2984
S	2.2860	0.5802	1.9679
H	-0.1774	-0.9178	3.3616
H	1.9276	0.6196	3.5744
C	1.2268	0.1800	5.6269
C	2.0241	0.9185	4.9787
C	0.2426	-0.7339	6.0374

C	0.5956	-2.0521	6.4606
C	-1.1354	-0.3540	6.0218
C	-0.3998	-2.9516	6.8484
H	1.6504	-2.3405	6.4807
C	-2.1181	-1.2694	6.4144
H	-1.4048	0.6580	5.7066
C	-1.7560	-2.5653	6.8271
H	-0.1233	-3.9588	7.1750
H	-3.1710	-0.9720	6.4024
H	-2.5291	-3.2742	7.1393
C	2.9795	2.0183	5.1370
C	2.6465	3.1280	5.9517
C	4.2396	1.9858	4.4945
C	3.5608	4.1769	6.1203
H	1.6715	3.1541	6.4475
C	5.1508	3.0387	4.6733
H	4.5068	1.1281	3.8702
C	4.8138	4.1374	5.4820
H	3.2940	5.0280	6.7549
H	6.1282	2.9968	4.1820
H	5.5249	4.9582	5.6177

Stationary point: ^{3}TS2 (s = 1)

Mo	0.5890	1.5871	0.4778
Mo	1.4681	-1.0664	0.5376
S	0.1819	-0.0791	-1.2015
Cl	1.1449	-3.2540	-0.7317
Cl	2.4383	2.4268	-1.1157
Mo	-1.3135	-0.5002	0.6106
Cl	-3.0446	0.6051	-0.9320
N	-3.1479	-1.1485	1.6686
C	-3.6146	-2.3733	1.3664
C	0.0359	4.5650	0.8257
N	0.8481	3.6157	1.3239
C	4.3349	-2.0694	0.8744
N	3.1222	-2.2459	1.4257
C	3.2600	-3.2156	2.4052
H	2.4136	-3.5567	2.9957
N	3.2450	-0.7582	-0.8359
H	3.2184	0.2263	-1.1662
H	3.1034	-1.3661	-1.6531
C	4.5659	-1.0547	-0.2018
H	4.9518	-0.1164	0.2359
H	5.3028	-1.4018	-0.9460
N	5.2475	-2.8868	1.4697

C	6.6782	-2.9620	1.1522
H	7.1346	-3.7303	1.7886
H	6.8199	-3.2402	0.0966
H	7.1647	-1.9946	1.3480
C	4.5789	-3.6186	2.4443
H	5.0961	-4.3496	3.0606
N	-0.6929	2.9484	-0.8155
H	-1.5499	2.4246	-1.0749
H	-0.1587	3.1303	-1.6755
C	-1.0521	4.2346	-0.1493
H	-2.0066	4.0822	0.3868
H	-1.2093	5.0428	-0.8835
N	0.3281	5.7746	1.3809
C	-0.3487	7.0465	1.1007
H	-0.3383	7.2503	0.0192
H	-1.3884	7.0172	1.4615
H	0.1917	7.8487	1.6192
C	1.6887	4.2387	2.2313
H	2.4492	3.6886	2.7784
C	1.3726	5.5806	2.2774
H	1.7906	6.4051	2.8490
N	-1.9930	-2.4216	-0.4116
H	-1.1526	-2.9480	-0.7162
H	-2.4978	-2.1218	-1.2566
C	-2.8769	-3.2800	0.4299
H	-3.5655	-3.8854	-0.1843
H	-2.2370	-3.9809	0.9957
N	-4.7723	-2.6169	2.0414
C	-5.5541	-3.8577	2.0078
H	-5.7262	-4.1657	0.9661
H	-5.0289	-4.6610	2.5477
H	-6.5237	-3.6711	2.4867
C	-5.0525	-1.4916	2.8087
C	-4.0408	-0.5865	2.5659
H	-3.8998	0.4171	2.9582
H	-5.9337	-1.4421	3.4432
S	-1.1760	1.3923	1.9874
S	-0.0899	-1.9577	2.1901
S	2.3457	0.7179	1.8765
H	0.0532	-1.1485	3.2867
H	1.9355	0.6536	3.4014
C	0.9000	0.4563	5.4115
C	1.9771	0.8263	4.8680
C	-0.3304	0.0047	5.9264
C	-0.5262	-1.3746	6.2437
C	-1.3809	0.9330	6.1990

C	-1.7320	-1.8000	6.8084
H	0.2818	-2.0870	6.0529
C	-2.5757	0.4902	6.7724
H	-1.2322	1.9913	5.9674
C	-2.7578	-0.8732	7.0766
H	-1.8702	-2.8574	7.0542
H	-3.3708	1.2101	6.9894
H	-3.6955	-1.2121	7.5274
C	3.2802	1.4393	5.1355
C	3.3468	2.6326	5.8959
C	4.4764	0.8542	4.6590
C	4.5891	3.2128	6.1876
H	2.4211	3.0881	6.2597
C	5.7158	1.4380	4.9639
H	4.4305	-0.0653	4.0682
C	5.7764	2.6178	5.7250
H	4.6291	4.1310	6.7823
H	6.6372	0.9674	4.6062
H	6.7448	3.0721	5.9568

MECP1

Mo	0.1976	1.3710	-1.1896
Mo	0.8636	-1.3211	-1.0630
S	-0.3405	-0.2864	-2.8346
Cl	0.3800	-3.5248	-2.2649
Cl	2.0401	2.0431	-2.8818
Mo	-1.8113	-0.4880	-0.9846
Cl	-3.4708	0.7098	-2.5054
N	-3.6920	-0.9613	0.0895
C	-4.2753	-2.1332	-0.2195
C	-0.0645	4.4011	-0.8858
N	0.6720	3.3887	-0.3931
C	3.6314	-2.5474	-0.6824
N	2.4099	-2.5985	-0.1234
C	2.4630	-3.5463	0.8848
H	1.5913	-3.7953	1.4851
N	2.6475	-1.2319	-2.4538
H	2.6976	-0.2720	-2.8439
H	2.4649	-1.8802	-3.2311
C	3.9380	-1.5816	-1.7849
H	4.3618	-0.6509	-1.3659
H	4.6683	-1.9876	-2.5043
N	4.4674	-3.4294	-0.0663
C	5.8892	-3.6393	-0.3630
H	6.2183	-4.5563	0.1425

H	6.0330	-3.7596	-1.4468
H	6.4880	-2.7890	-0.0021
C	3.7393	-4.0655	0.9328
H	4.1896	-4.8233	1.5694
N	-0.9824	2.8352	-2.4712
H	-1.8856	2.3828	-2.7108
H	-0.4489	2.9639	-3.3410
C	-1.2171	4.1509	-1.8075
H	-2.1568	4.0748	-1.2307
H	-1.3434	4.9606	-2.5456
N	0.3750	5.5900	-0.3875
C	-0.1897	6.9150	-0.6668
H	-0.3107	7.0505	-1.7516
H	-1.1644	7.0324	-0.1686
H	0.5052	7.6763	-0.2895
C	1.6155	3.9493	0.4511
H	2.3448	3.3438	0.9842
C	1.4370	5.3166	0.4664
H	1.9631	6.1106	0.9902
N	-2.6494	-2.3560	-1.9859
H	-1.8517	-2.9616	-2.2606
H	-3.1082	-2.0355	-2.8487
C	-3.6200	-3.1137	-1.1425
H	-4.3550	-3.6572	-1.7597
H	-3.0550	-3.8656	-0.5624
N	-5.4566	-2.2607	0.4458
C	-6.3642	-3.4126	0.3938
H	-6.5689	-3.6819	-0.6529
H	-5.9236	-4.2746	0.9179
H	-7.3080	-3.1351	0.8803
C	-5.6287	-1.1150	1.2147
C	-4.5306	-0.3138	0.9810
H	-4.2939	0.6704	1.3770
H	-6.5064	-0.9774	1.8405
S	-1.5095	1.3808	0.4061
S	-0.7552	-1.9959	0.6394
S	1.8426	0.3956	0.1810
H	-0.5779	-1.1607	1.7257
H	1.7297	0.4408	2.4386
C	0.8553	0.1505	4.2789
C	1.6979	0.8022	3.5157
C	-0.1462	-0.7591	4.5820
C	0.1570	-2.0493	5.1465
C	-1.5236	-0.4394	4.3062
C	-0.8647	-2.9602	5.4027
H	1.1993	-2.2993	5.3643

C	-2.5294	-1.3706	4.5747
H	-1.7637	0.5437	3.8915
C	-2.2106	-2.6292	5.1229
H	-0.6226	-3.9390	5.8288
H	-3.5711	-1.1169	4.3557
H	-3.0058	-3.3492	5.3389
C	2.6095	1.9307	3.7943
C	2.3866	2.8271	4.8646
C	3.7340	2.1272	2.9581
C	3.2684	3.8931	5.0882
H	1.5106	2.6847	5.5063
C	4.6198	3.1905	3.1916
H	3.9127	1.4343	2.1285
C	4.3872	4.0792	4.2547
H	3.0801	4.5866	5.9142
H	5.4904	3.3239	2.5409
H	5.0722	4.9143	4.4323

Stationary point: ^{1}I2 (s = 0)

Mo	-0.0697	1.2960	-1.2734
Mo	1.3107	-1.0751	-1.4864
S	-0.2943	-0.2914	-3.0533
Cl	1.3496	-3.2231	-2.8877
Cl	1.3605	2.6006	-2.9944
Mo	-1.4560	-1.0556	-1.1281
Cl	-3.5325	-0.2702	-2.3908
N	-3.0031	-2.1440	0.0343
C	-3.2498	-3.4117	-0.3412
C	-1.0789	4.1065	-0.6190
N	-0.0609	3.2883	-0.2991
C	4.3410	-1.4647	-1.4455
N	3.2464	-1.9307	-0.8199
C	3.6667	-2.9294	0.0423
H	2.9666	-3.4887	0.6576
N	2.8536	-0.3384	-2.9728
H	2.5837	0.6258	-3.2482
H	2.7885	-0.9358	-3.8074
C	4.2490	-0.3498	-2.4404
H	4.4287	0.6210	-1.9439
H	4.9912	-0.4529	-3.2496
N	5.4501	-2.1261	-1.0111
C	6.8342	-1.8786	-1.4311
H	7.4651	-2.6897	-1.0456
H	6.8987	-1.8691	-2.5291
H	7.1919	-0.9173	-1.0313

C	5.0366	-3.0554	-0.0636
H	5.7442	-3.7155	0.4313
N	-1.7259	2.4918	-2.2907
H	-2.4976	1.8311	-2.5034
H	-1.3472	2.8348	-3.1827
C	-2.2212	3.6325	-1.4650
H	-3.0373	3.2537	-0.8231
H	-2.6366	4.4387	-2.0925
N	-0.9009	5.3272	-0.0395
C	-1.8070	6.4782	-0.1267
H	-2.0791	6.6645	-1.1761
H	-2.7176	6.2991	0.4653
H	-1.2862	7.3611	0.2659
C	0.7994	4.0080	0.5118
H	1.7183	3.5740	0.8976
C	0.2849	5.2762	0.6842
H	0.6480	6.1368	1.2398
N	-1.8328	-3.0088	-2.2502
H	-0.9293	-3.3281	-2.6516
H	-2.4588	-2.7772	-3.0326
C	-2.4417	-4.0808	-1.4094
H	-3.0482	-4.7767	-2.0134
H	-1.6218	-4.6675	-0.9564
N	-4.2582	-3.9374	0.4089
C	-4.7961	-5.2996	0.3164
H	-5.0381	-5.5386	-0.7299
H	-4.0684	-6.0292	0.7037
H	-5.7151	-5.3518	0.9143
C	-4.6662	-2.9562	1.3056
C	-3.8840	-1.8473	1.0609
H	-3.8994	-0.8700	1.5365
H	-5.4684	-3.1356	2.0167
S	-1.5498	0.6904	0.4335
S	0.1410	-2.2370	0.2921
S	1.8241	0.6828	-0.0246
H	0.2563	-1.3429	1.3603
H	0.7204	0.4023	2.4265
C	0.0149	1.3066	4.1553
C	0.8544	0.5107	3.5527
C	-0.9628	2.1512	4.6360
C	-2.2282	1.6253	5.0863
C	-0.7405	3.5737	4.7127
C	-3.2094	2.4863	5.5709
H	-2.3959	0.5456	5.0446
C	-1.7400	4.4110	5.2006
H	0.2197	3.9767	4.3796

C	-2.9747	3.8773	5.6304
H	-4.1669	2.0798	5.9102
H	-1.5647	5.4898	5.2533
H	-3.7517	4.5445	6.0150
C	1.9779	-0.2678	4.1282
C	2.2050	-0.3008	5.5249
C	2.8429	-0.9891	3.2768
C	3.2724	-1.0382	6.0508
H	1.5353	0.2549	6.1901
C	3.9130	-1.7266	3.8086
H	2.6795	-0.9607	2.1938
C	4.1317	-1.7543	5.1958
H	3.4358	-1.0563	7.1332
H	4.5745	-2.2782	3.1325
H	4.9654	-2.3298	5.6105

Stationary point: ^{3}I2 (s = 1)

Mo	0.1643	1.6146	-2.2016
Mo	0.9885	-1.0913	-2.1622
S	-0.2056	-0.0027	-3.9031
Cl	0.7517	-3.1392	-3.6524
Cl	2.1336	2.4006	-3.6063
Mo	-1.7101	-0.4276	-2.0788
Cl	-3.4512	0.6982	-3.6100
N	-3.5494	-1.0911	-1.0499
C	-4.0643	-2.2669	-1.4527
C	-0.3587	4.6016	-1.8464
N	0.4356	3.6386	-1.3464
C	3.7847	-2.2275	-1.6459
N	2.5202	-2.3758	-1.2116
C	2.5302	-3.4008	-0.2794
H	1.6227	-3.7313	0.2193
N	2.9042	-0.7720	-3.3548
H	2.9483	0.2223	-3.6494
H	2.7975	-1.3422	-4.2044
C	4.1519	-1.1645	-2.6342
H	4.5421	-0.2736	-2.1103
H	4.9333	-1.5019	-3.3356
N	4.6036	-3.1212	-1.0256
C	6.0550	-3.2442	-1.2041
H	6.3820	-4.1913	-0.7562
H	6.3017	-3.2539	-2.2759
H	6.5762	-2.4087	-0.7121
C	3.8208	-3.8685	-0.1526
H	4.2493	-4.6560	0.4619

N	-1.0620	2.9976	-3.5061
H	-1.9167	2.4837	-3.7944
H	-0.5122	3.1916	-4.3536
C	-1.4376	4.2781	-2.8325
H	-2.3963	4.1131	-2.3082
H	-1.5927	5.0880	-3.5646
N	-0.0484	5.8041	-1.2867
C	-0.7164	7.0865	-1.5387
H	-0.8492	7.2344	-2.6202
H	-1.6966	7.1155	-1.0383
H	-0.0828	7.8921	-1.1455
C	1.2850	4.2435	-0.4354
H	2.0444	3.6819	0.1024
C	0.9888	5.5894	-0.3861
H	1.4185	6.4049	0.1896
N	-2.4467	-2.2514	-3.2449
H	-1.6178	-2.7820	-3.5707
H	-2.9381	-1.8942	-4.0748
C	-3.3520	-3.1315	-2.4478
H	-4.0547	-3.6828	-3.0948
H	-2.7257	-3.8767	-1.9253
N	-5.2375	-2.5121	-0.8048
C	-6.0776	-3.7078	-0.9397
H	-6.1575	-3.9892	-1.9995
H	-5.6515	-4.5460	-0.3670
H	-7.0801	-3.4759	-0.5574
C	-5.4726	-1.4437	0.0527
C	-4.4219	-0.5654	-0.1120
H	-4.2446	0.3980	0.3590
H	-6.3568	-1.4055	0.6835
S	-1.6044	1.4569	-0.6954
S	-0.5942	-1.8855	-0.6364
S	1.8246	0.6759	-0.6546
H	-1.1367	-0.1952	1.9582
H	1.1433	0.4413	0.5223
C	-0.6040	-0.1966	2.9266
C	0.6636	0.1853	2.9508
C	-1.4222	-0.6503	4.0812
C	-0.9092	-0.6971	5.3991
C	-2.7603	-1.0502	3.8586
C	-1.7134	-1.1323	6.4598
H	0.1254	-0.3880	5.5832
C	-3.5638	-1.4870	4.9236
H	-3.1688	-1.0155	2.8417
C	-3.0439	-1.5299	6.2279
H	-1.3021	-1.1622	7.4743

H	-4.5979	-1.7931	4.7333
H	-3.6694	-1.8696	7.0596
C	1.9650	0.5243	3.2940
C	2.3109	1.8749	3.6634
C	3.0245	-0.4538	3.2510
C	3.6288	2.2083	3.9724
H	1.5192	2.6288	3.7063
C	4.3336	-0.0933	3.5669
H	2.7802	-1.4839	2.9754
C	4.6505	1.2348	3.9268
H	3.8692	3.2374	4.2594
H	5.1215	-0.8534	3.5401
H	5.6812	1.5070	4.1731

Stationary point: $^{3}TS_{3\text{-}cis}$ (s = 1)

Mo	0.5152	1.7322	0.3608
Mo	1.3701	-0.9627	0.4651
S	0.2097	0.0843	-1.3239
Cl	1.2053	-3.0424	-1.0018
Cl	2.4960	2.5317	-1.0296
Mo	-1.3379	-0.3429	0.4624
Cl	-3.0544	0.7313	-1.1405
N	-3.1981	-1.0247	1.4416
C	-3.6804	-2.2144	1.0395
C	-0.0604	4.7166	0.6471
N	0.7308	3.7735	1.1881
C	4.1673	-2.0555	1.0602
N	2.8968	-2.2101	1.4740
C	2.9012	-3.2155	2.4274
H	1.9880	-3.5493	2.9135
N	3.3059	-0.6448	-0.6975
H	3.3423	0.3460	-1.0043
H	3.2248	-1.2286	-1.5404
C	4.5417	-1.0083	0.0575
H	4.9083	-0.1024	0.5725
H	5.3422	-1.3500	-0.6198
N	4.9850	-2.9267	1.7142
C	6.4413	-3.0351	1.5664
H	6.7698	-3.9740	2.0302
H	6.7096	-3.0523	0.4999
H	6.9431	-2.1888	2.0603
C	4.1950	-3.6650	2.5880
H	4.6205	-4.4351	3.2261
N	-0.7086	3.0722	-0.9948
H	-1.5496	2.5409	-1.2915

H	-0.1420	3.2567	-1.8333
C	-1.1141	4.3600	-0.3547
H	-2.0810	4.1930	0.1535
H	-1.2650	5.1541	-1.1049
N	0.2270	5.9355	1.1833
C	-0.4412	7.2061	0.8777
H	-0.5355	7.3279	-0.2114
H	-1.4390	7.2390	1.3414
H	0.1720	8.0256	1.2743
C	1.5535	4.4087	2.1032
H	2.3062	3.8676	2.6708
C	1.2449	5.7527	2.1124
H	1.6544	6.5860	2.6774
N	-2.0090	-2.1937	-0.7019
H	-1.1619	-2.7139	-0.9966
H	-2.4822	-1.8541	-1.5498
C	-2.9219	-3.0796	0.0795
H	-3.5940	-3.6530	-0.5806
H	-2.2985	-3.8058	0.6313
N	-4.8715	-2.4686	1.6503
C	-5.6887	-3.6788	1.5054
H	-5.7490	-3.9644	0.4452
H	-5.2572	-4.5082	2.0868
H	-6.7004	-3.4629	1.8725
C	-5.1531	-1.3909	2.4819
C	-4.1113	-0.4986	2.3395
H	-3.9651	0.4754	2.7996
H	-6.0588	-1.3579	3.0817
S	-1.3032	1.5606	1.8163
S	-0.2393	-1.7618	1.9520
S	2.1857	0.8394	1.9213
H	-0.6577	0.5230	4.2282
H	1.6217	0.6254	3.2327
C	-0.0291	0.2970	5.1081
C	1.2871	0.4875	5.0018
C	-0.7721	-0.2303	6.2787
C	-0.1378	-0.5964	7.4902
C	-2.1776	-0.3682	6.1810
C	-0.8908	-1.0762	8.5694
H	0.9486	-0.5008	7.5818
C	-2.9291	-0.8486	7.2642
H	-2.6788	-0.0933	5.2460
C	-2.2886	-1.2042	8.4629
H	-0.3852	-1.3536	9.5003
H	-4.0159	-0.9445	7.1709
H	-2.8724	-1.5803	9.3091

C	2.5526	0.5810	5.6254
C	2.8898	1.7285	6.4175
C	3.5553	-0.4233	5.4236
C	4.1562	1.8422	6.9946
H	2.1348	2.5054	6.5708
C	4.8140	-0.2941	6.0148
H	3.3159	-1.2992	4.8121
C	5.1245	0.8360	6.7989
H	4.3942	2.7185	7.6060
H	5.5635	-1.0782	5.8666
H	6.1150	0.9324	7.2539

Stationary point: $^{3}TS_{3\text{-}trans}$ (s = 1)

Mo	0.5637	1.1947	0.0690
Mo	1.9885	-1.2030	0.5316
S	0.8938	-0.5773	-1.4774
Cl	2.4907	-3.3532	-0.7748
Cl	2.4843	2.3492	-1.1668
Mo	-0.7614	-1.2549	0.1210
Cl	-2.4276	-0.7307	-1.7835
N	-2.5277	-2.3129	0.9322
C	-2.6593	-3.6056	0.5849
C	-0.7008	3.9712	-0.0194
N	0.2056	3.2864	0.6997
C	4.8584	-1.6693	1.4877
N	3.6024	-2.0440	1.7941
C	3.6993	-3.0351	2.7570
H	2.8183	-3.5190	3.1707
N	3.9455	-0.5215	-0.4252
H	3.8174	0.4408	-0.7918
H	4.0899	-1.1421	-1.2328
C	5.1275	-0.5908	0.4846
H	5.2285	0.3862	0.9900
H	6.0599	-0.7714	-0.0759
N	5.7550	-2.3852	2.2220
C	7.2167	-2.2545	2.1828
H	7.6555	-3.0858	2.7494
H	7.5705	-2.3066	1.1426
H	7.5315	-1.3007	2.6327
C	5.0320	-3.2519	3.0343
H	5.5274	-3.9327	3.7214
N	-0.7202	2.1154	-1.5571
H	-1.3607	1.3820	-1.9165
H	-0.0929	2.3908	-2.3246
C	-1.5056	3.2966	-1.0862

H	-2.4561	2.9200	-0.6668
H	-1.7516	3.9769	-1.9184
N	-0.7527	5.2664	0.3998
C	-1.6448	6.3167	-0.1046
H	-1.7001	6.2648	-1.2014
H	-2.6539	6.2044	0.3211
H	-1.2329	7.2926	0.1837
C	0.7565	4.1728	1.6095
H	1.5409	3.8724	2.2995
C	0.1623	5.4057	1.4373
H	0.3089	6.3576	1.9407
N	-0.8151	-3.2807	-0.9386
H	0.1663	-3.5914	-1.0678
H	-1.2205	-3.1153	-1.8692
C	-1.6044	-4.3150	-0.2085
H	-2.0360	-5.0584	-0.8997
H	-0.9167	-4.8527	0.4692
N	-3.8127	-4.1157	1.1001
C	-4.2845	-5.4998	0.9785
H	-4.1690	-5.8438	-0.0597
H	-3.7173	-6.1613	1.6516
H	-5.3484	-5.5318	1.2468
C	-4.4379	-3.0985	1.8123
C	-3.6339	-1.9839	1.6973
H	-3.7758	-0.9830	2.0970
H	-5.3859	-3.2597	2.3189
S	-1.3720	0.6826	1.2585
S	0.4043	-2.2657	1.8691
S	2.1583	0.8319	1.8949
H	-0.1676	-0.7070	5.8593
H	1.5425	0.6224	3.2113
C	0.0340	0.2261	5.2997
C	1.3247	0.4859	4.9840
C	-1.1538	1.0224	4.9849
C	-1.0590	2.3338	4.4527
C	-2.4372	0.5095	5.2991
C	-2.2094	3.1008	4.2408
H	-0.0716	2.7430	4.2149
C	-3.5892	1.2793	5.0829
H	-2.5219	-0.4963	5.7262
C	-3.4797	2.5776	4.5540
H	-2.1179	4.1143	3.8368
H	-4.5725	0.8709	5.3379
H	-4.3774	3.1843	4.3968
C	2.5837	0.3147	5.6281
C	2.6611	0.5287	7.0455

C	3.7959	0.0085	4.9346
C	3.8775	0.4207	7.7218
H	1.7438	0.7799	7.5867
C	5.0064	-0.0965	5.6246
H	3.7685	-0.1539	3.8539
C	5.0577	0.1096	7.0177
H	3.9107	0.5803	8.8044
H	5.9213	-0.3406	5.0760
H	6.0110	0.0322	7.5495

MECP2

Mo	0.0915	1.6143	-2.1898
Mo	0.9433	-1.0758	-2.0880
S	-0.2303	-0.0351	-3.8694
Cl	0.7627	-3.1589	-3.5437
Cl	2.0684	2.4085	-3.5858
Mo	-1.7603	-0.4586	-2.0703
Cl	-3.4856	0.6230	-3.6531
N	-3.6161	-1.1383	-1.0760
C	-4.1015	-2.3284	-1.4734
C	-0.4865	4.5975	-1.8927
N	0.3117	3.6544	-1.3622
C	3.7402	-2.1718	-1.4911
N	2.4695	-2.3270	-1.0782
C	2.4746	-3.3272	-0.1194
H	1.5626	-3.6614	0.3683
N	2.8789	-0.7662	-3.2553
H	2.9180	0.2222	-3.5699
H	2.7971	-1.3543	-4.0951
C	4.1144	-1.1276	-2.4979
H	4.4788	-0.2198	-1.9843
H	4.9168	-1.4708	-3.1726
N	4.5591	-3.0362	-0.8299
C	6.0161	-3.1426	-0.9706
H	6.3424	-4.0831	-0.5079
H	6.2902	-3.1566	-2.0355
H	6.5141	-2.2972	-0.4714
C	3.7697	-3.7717	0.0469
H	4.1969	-4.5359	0.6907
N	-1.1443	2.9540	-3.5316
H	-1.9873	2.4231	-3.8244
H	-0.5840	3.1401	-4.3736
C	-1.5453	4.2427	-2.8898
H	-2.5103	4.0782	-2.3766
H	-1.6973	5.0370	-3.6396

N	-0.2055	5.8129	-1.3461
C	-0.8847	7.0806	-1.6385
H	-0.9878	7.2080	-2.7259
H	-1.8798	7.1028	-1.1681
H	-0.2751	7.9024	-1.2409
C	1.1318	4.2862	-0.4423
H	1.8865	3.7439	0.1212
C	0.8151	5.6281	-0.4204
H	1.2211	6.4593	0.1508
N	-2.4395	-2.3124	-3.2231
H	-1.5928	-2.8323	-3.5203
H	-2.9151	-1.9751	-4.0701
C	-3.3500	-3.1959	-2.4362
H	-4.0261	-3.7678	-3.0941
H	-2.7268	-3.9250	-1.8879
N	-5.2907	-2.5798	-0.8584
C	-6.1104	-3.7890	-0.9991
H	-6.1786	-4.0741	-2.0589
H	-5.6770	-4.6192	-0.4203
H	-7.1192	-3.5709	-0.6257
C	-5.5685	-1.4997	-0.0285
C	-4.5259	-0.6092	-0.1761
H	-4.3787	0.3666	0.2798
H	-6.4728	-1.4645	0.5737
S	-1.7029	1.4379	-0.7098
S	-0.6455	-1.8543	-0.5774
S	1.7779	0.7277	-0.6389
H	-1.0872	0.4207	1.8021
H	1.1707	0.5062	0.6015
C	-0.4804	0.1773	2.6937
C	0.8312	0.3859	2.6465
C	-1.2492	-0.3744	3.8385
C	-0.6304	-0.7482	5.0553
C	-2.6511	-0.5185	3.7178
C	-1.3950	-1.2424	6.1193
H	0.4542	-0.6441	5.1603
C	-3.4152	-1.0126	4.7867
H	-3.1411	-0.2377	2.7784
C	-2.7905	-1.3753	5.9917
H	-0.9006	-1.5249	7.0546
H	-4.5002	-1.1115	4.6789
H	-3.3850	-1.7593	6.8269
C	2.1208	0.4877	3.1893
C	2.5039	1.6480	3.9471
C	3.1105	-0.5293	2.9626
C	3.7942	1.7595	4.4656

H	1.7623	2.4335	4.1209
C	4.3943	-0.3975	3.4939
H	2.8379	-1.4135	2.3780
C	4.7468	0.7421	4.2472
H	4.0651	2.6438	5.0515
H	5.1315	-1.1892	3.3253
H	5.7552	0.8360	4.6613

Stationary point: [$Mo_3S_4Cl_3(dmen)_3]^+$

S	-0.1981	-2.0645	-0.1981
Mo	-1.3812	-0.4173	0.9655
S	0.3388	1.1371	1.1042
Mo	-0.9242	1.7297	-0.7705
S	-2.9345	0.4677	-0.6011
Mo	-1.3479	-0.8348	-1.8024
Cl	-2.5618	-0.1931	-3.9256
N	0.5197	3.4924	-1.1184
C	0.2565	4.0870	-2.4657
C	-1.2413	4.2302	-2.6889
N	-1.9391	2.9200	-2.4993
C	-3.4182	3.1181	-2.5194
C	1.9843	3.3405	-0.8859
Cl	-2.0509	3.5502	0.5826
S	0.3806	0.6391	-2.3525
N	-0.4590	-2.3540	-3.2939
C	1.0156	-2.5181	-3.4383
N	-2.9476	-2.5395	-1.8593
C	-4.4043	-2.2161	-1.8705
C	-1.1208	-3.6814	-3.1008
C	-2.6236	-3.4976	-2.9621
N	-0.5015	-1.2292	2.9392
C	-0.7833	-0.2556	4.0390
C	-2.2268	0.2148	3.9568
N	-2.5246	0.7898	2.6081
C	-3.9867	1.0568	2.4750
Cl	-3.1284	-2.1355	1.5999
C	0.9073	-1.7110	3.0084
H	-0.8180	-1.9569	-4.1770
H	-2.7611	-3.0026	-0.9548
H	-1.1190	-2.0446	3.0840
H	-2.0540	1.7063	2.5266
H	0.1567	4.1529	-0.4122
H	-1.7178	2.3082	-3.3020
H	1.4648	-1.5541	-3.7133
H	1.2430	-3.2644	-4.2196

H	1.4405	-2.8530	-2.4804
H	-0.6871	-4.1411	-2.1967
H	-0.9017	-4.3454	-3.9571
H	-3.0453	-3.0753	-3.8896
H	-3.1127	-4.4712	-2.7788
H	-4.6616	-1.6894	-0.9418
H	-4.9988	-3.1444	-1.9386
H	-4.6204	-1.5735	-2.7362
H	1.0569	-2.5067	2.2660
H	1.1295	-2.1022	4.0167
H	1.5889	-0.8771	2.7838
H	-0.5948	-0.7245	5.0223
H	-0.0822	0.5883	3.9249
H	-2.4326	0.9615	4.7446
H	-2.9163	-0.6334	4.1038
H	-4.1583	1.6739	1.5829
H	-4.3564	1.5953	3.3655
H	-4.5162	0.0983	2.3743
H	2.1575	3.0145	0.1488
H	2.5020	4.3000	-1.0604
H	2.3850	2.5807	-1.5730
H	0.7106	3.4201	-3.2183
H	0.7448	5.0753	-2.5494
H	-1.6733	4.9329	-1.9566
H	-1.4410	4.6270	-3.7004
H	-3.9093	2.1395	-2.6042
H	-3.7052	3.7453	-3.3818
H	-3.7242	3.6091	-1.5845

Stationary point: $^{1}TS_2$ – $[Mo_3S_4Cl_3(dmen)_3]^+$ (s = 0)

Mo	0.8271	1.3719	0.3240
Mo	1.4242	-1.3341	0.6498
S	0.4270	-0.3595	-1.2727
Cl	0.9546	-3.6239	-0.3767
Cl	2.9475	1.8872	-1.0346
Mo	-1.1908	-0.4221	0.4625
Cl	-2.7407	0.7919	-1.1324
N	-3.2878	-0.6792	1.4613
N	1.4418	3.4906	1.0912
N	2.8106	-2.7740	1.8509
N	3.3860	-1.2995	-0.5561
C	4.4902	-1.7910	0.3288
H	4.7132	-1.0016	1.0648
H	5.4063	-1.9772	-0.2611
N	-0.2308	2.7826	-1.1374

C	-0.3217	4.1397	-0.5074
H	-1.0796	4.0927	0.2918
H	-0.6522	4.8880	-1.2503
N	-1.9977	-2.3758	-0.4780
C	-3.0891	-2.9039	0.4021
H	-3.6305	-3.7259	-0.1012
H	-2.6243	-3.3130	1.3142
S	-0.9539	1.4928	1.8416
S	-0.3092	-1.8016	2.2736
S	2.3287	0.4376	1.9644
H	-0.1839	-0.8069	3.2293
H	1.8967	0.5509	3.5496
C	1.1837	0.1617	5.6340
C	1.9758	0.8877	4.9664
C	0.2305	-0.7360	6.1438
C	0.6130	-2.0468	6.5647
C	-1.1376	-0.3397	6.2599
C	-0.3473	-2.9277	7.0672
H	1.6633	-2.3444	6.4963
C	-2.0837	-1.2341	6.7702
H	-1.4272	0.6699	5.9553
C	-1.6947	-2.5266	7.1707
H	-0.0485	-3.9300	7.3886
H	-3.1289	-0.9235	6.8618
H	-2.4399	-3.2201	7.5724
C	2.9218	1.9980	5.1239
C	2.6082	3.0558	6.0142
C	4.1498	2.0293	4.4228
C	3.5079	4.1144	6.1956
H	1.6586	3.0337	6.5571
C	5.0479	3.0907	4.6165
H	4.4053	1.2163	3.7376
C	4.7295	4.1369	5.4983
H	3.2546	4.9241	6.8874
H	6.0010	3.0959	4.0781
H	5.4301	4.9649	5.6449
C	4.0351	-3.0644	1.0276
H	3.7676	-3.8346	0.2871
H	4.8374	-3.4725	1.6674
C	-4.0416	-1.7676	0.7489
H	-4.4713	-1.3356	-0.1687
H	-4.8751	-2.1298	1.3762
C	1.0414	4.5091	0.0602
H	1.8024	4.5040	-0.7358
H	1.0224	5.5186	0.5076
C	3.4323	-1.9414	-1.9018

H	2.6625	-1.4865	-2.5403
H	4.4263	-1.7937	-2.3604
H	3.2191	-3.0159	-1.8155
H	3.5207	-0.2803	-0.7107
C	3.1614	-2.4859	3.2696
H	3.8017	-1.5937	3.3193
H	2.2425	-2.2963	3.8410
H	3.6994	-3.3417	3.7140
H	2.2358	-3.6281	1.8433
C	-2.3816	-2.4116	-1.9190
H	-3.2488	-1.7604	-2.0945
H	-1.5406	-2.0413	-2.5222
H	-2.6281	-3.4452	-2.2203
C	-3.4045	-0.8095	2.9411
H	-2.9825	-1.7730	3.2618
H	-2.8409	0.0026	3.4221
H	-4.4636	-0.7582	3.2498
H	-3.7234	0.2144	1.1960
H	-1.1703	-3.0015	-0.3904
C	1.0718	3.9416	2.4619
H	-0.0186	4.0680	2.5254
H	1.3824	3.1860	3.1949
H	1.5643	4.9026	2.6930
C	0.1850	2.8631	-2.5672
H	1.1926	3.2944	-2.6409
H	0.2072	1.8498	-2.9913
H	-0.5297	3.4869	-3.1320
H	-1.1804	2.3545	-1.1352
H	2.4675	3.4103	1.0556

Stationary point: $^{3}I_2$ – $[Mo_3S_4Cl_3(dmen)_3]^+$ (s = 1)

Mo	0,6158	2,2210	-2,1422
Mo	1,2912	-0,5189	-2,5155
S	0,3998	0,9597	-4,1463
Cl	1,1100	-2,2967	-4,3026
Cl	2,8400	3,0832	-2,9810
Mo	-1,3662	0,3305	-2,6451
Cl	-2,8694	1,8835	-3,9996
N	-3,4788	-0,2666	-1,9013
N	1,0086	4,0640	-0,7765
N	2,5413	-2,2588	-1,6184
N	3,4259	-0,0933	-3,2921
H	3,5392	0,9170	-3,0795
C	4,4032	-0,8712	-2,4632
H	4,5009	-0,3697	-1,4865

H	5,3990	-0,8761	-2,9419
N	-0,4031	3,9181	-3,2849
H	-1,3184	3,4844	-3,5229
C	-0,6538	5,0437	-2,3260
H	-1,4815	4,7447	-1,6618
H	-0,9648	5,9521	-2,8724
N	-2,0181	-1,2758	-4,1811
H	-1,1630	-1,8638	-4,2096
C	-3,1255	-2,0738	-3,5612
H	-3,6073	-2,7198	-4,3173
H	-2,6823	-2,7242	-2,7894
S	-1,3269	1,9059	-0,9145
S	-0,5212	-1,4170	-1,3503
S	1,9754	0,8706	-0,5952
H	-1,1912	-0,3974	2,1476
H	1,1042	0,4852	0,3970
C	-0,6509	-0,3513	3,1095
C	0,6199	0,0204	3,1090
C	-1,4653	-0,7268	4,2948
C	-0,9371	-0,7131	5,6075
C	-2,8136	-1,1098	4,1108
C	-1,7368	-1,0714	6,6998
H	0,1063	-0,4179	5,7628
C	-3,6134	-1,4688	5,2068
H	-3,2336	-1,1244	3,0985
C	-3,0786	-1,4510	6,5056
H	-1,3131	-1,0550	7,7095
H	-4,6558	-1,7627	5,0448
H	-3,7009	-1,7302	7,3619
C	1,9246	0,3660	3,4272
C	2,2825	1,7306	3,7298
C	2,9766	-0,6215	3,4362
C	3,6037	2,0693	4,0181
H	1,4960	2,4909	3,7438
C	4,2896	-0,2552	3,7282
H	2,7220	-1,6641	3,2240
C	4,6186	1,0874	4,0169
H	3,8518	3,1091	4,2559
H	5,0710	-1,0223	3,7404
H	5,6520	1,3637	4,2464
C	-4,1431	-1,1219	-2,9472
H	-4,5502	-0,4500	-3,7190
H	-4,9853	-1,6804	-2,5027
C	0,6167	5,3063	-1,5288
H	1,4461	5,5617	-2,2071
H	0,4774	6,1493	-0,8299

C	3,8851	-2,2926	-2,2913
H	3,7523	-2,7728	-3,2736
H	4,5929	-2,9033	-1,7038
H	2,0127	-3,0712	-1,9646
H	2,0372	4,0510	-0,7365
H	-3,9536	0,6462	-1,9183
C	0,1808	4,4050	-4,5701
H	0,2897	3,5558	-5,2589
H	-0,4827	5,1637	-5,0202
H	1,1733	4,8408	-4,3920
C	3,7058	-0,2267	-4,7535
H	3,0073	0,4135	-5,3106
H	4,7432	0,0840	-4,9691
H	3,5569	-1,2672	-5,0717
C	-2,3140	-0,9138	-5,5977
H	-3,2078	-0,2775	-5,6454
H	-1,4630	-0,3528	-6,0090
H	-2,4771	-1,8292	-6,1932
C	0,5117	4,0902	0,6290
H	0,9282	4,9617	1,1636
H	-0,5856	4,1539	0,6351
H	0,8151	3,1668	1,1417
C	-3,6552	-0,8311	-0,5325
H	-3,2032	-1,8321	-0,4827
H	-3,1510	-0,1789	0,1940
H	-4,7285	-0,9041	-0,2842
C	2,6567	-2,3990	-0,1384
H	3,1473	-3,3536	0,1202
H	3,2494	-1,5670	0,2689
H	1,6520	-2,3695	0,3068

Proton transfer

Stationary point: semihydrogenated dpa (dpaH)

H	-0.2495	0.5513	4.6643
C	0.1197	0.2345	5.6567
C	1.4021	0.4240	5.8165
C	-0.8776	-0.3275	6.5895
C	-0.5213	-0.7604	7.8888
C	-2.2190	-0.4289	6.1577
C	-1.5005	-1.2864	8.7382
H	0.5167	-0.6838	8.2275
C	-3.1926	-0.9577	7.0169
H	-2.4935	-0.0940	5.1525
C	-2.8366	-1.3866	8.3061
H	-1.2215	-1.6201	9.7423

H	-4.2298	-1.0337	6.6769
H	-3.5977	-1.7989	8.9756
C	2.7472	0.6354	5.9447
C	3.2331	1.8920	6.4660
C	3.6786	-0.4018	5.5654
C	4.6005	2.0889	6.5975
H	2.5115	2.6629	6.7471
C	5.0405	-0.1764	5.7083
H	3.2900	-1.3446	5.1727
C	5.4957	1.0608	6.2205
H	4.9875	3.0325	6.9899
H	5.7606	-0.9485	5.4269
H	6.5720	1.2275	6.3281

Stationary point: hydrosulfido cluster.

Mo	0.5255	1.6636	0.3333
Mo	1.3337	-0.9434	0.4638
S	0.1925	0.0411	-1.3741
Cl	1.1397	-3.1309	-0.9562
Cl	2.5129	2.4866	-1.1503
Mo	-1.3272	-0.3377	0.4371
Cl	-3.0730	0.7695	-1.1829
N	-3.2082	-1.0144	1.4211
C	-3.6913	-2.2010	1.0163
C	0.0127	4.6668	0.6499
N	0.7982	3.7116	1.1741
C	4.1564	-1.9955	1.0482
N	2.8971	-2.1648	1.4841
C	2.9398	-3.1336	2.4719
H	2.0388	-3.4645	2.9827
N	3.2436	-0.6884	-0.7611
H	3.2496	0.2889	-1.1113
H	3.1690	-1.3202	-1.5690
C	4.4905	-0.9754	0.0027
H	4.8206	-0.0359	0.4823
H	5.3100	-1.3129	-0.6553
N	5.0064	-2.8226	1.7219
C	6.4611	-2.9032	1.5544
H	6.8225	-3.8037	2.0678
H	6.7134	-2.9784	0.4862
H	6.9509	-2.0166	1.9864
C	4.2459	-3.5475	2.6331
H	4.6996	-4.2832	3.2923
N	-0.6832	3.0528	-1.0047
H	-1.5333	2.5349	-1.2966

H	-0.1156	3.2398	-1.8415
C	-1.0608	4.3328	-0.3402
H	-2.0169	4.1652	0.1886
H	-1.2211	5.1448	-1.0701
N	0.3100	5.8793	1.2009
C	-0.3559	7.1557	0.9209
H	-0.4718	7.2892	-0.1647
H	-1.3450	7.1920	1.4036
H	0.2689	7.9691	1.3127
C	1.6276	4.3304	2.0932
H	2.3754	3.7733	2.6524
C	1.3306	5.6775	2.1233
H	1.7479	6.5009	2.6971
N	-2.0072	-2.1973	-0.7141
H	-1.1435	-2.7110	-0.9773
H	-2.4654	-1.8716	-1.5743
C	-2.9262	-3.0726	0.0673
H	-3.5933	-3.6580	-0.5883
H	-2.3049	-3.7887	0.6356
N	-4.8787	-2.4640	1.6350
C	-5.6923	-3.6751	1.4883
H	-5.7803	-3.9415	0.4248
H	-5.2429	-4.5147	2.0413
H	-6.6954	-3.4732	1.8864
C	-5.1554	-1.3926	2.4770
C	-4.1142	-0.4988	2.3312
H	-3.9587	0.4691	2.8016
H	-6.0572	-1.3656	3.0832
S	-1.2661	1.5221	1.8792
S	-0.2692	-1.6912	2.0438
S	1.9520	0.7658	2.0584
H	1.0015	0.5217	3.0809

Hydrogen atom transfer

Stationary point: semihydrogenated dpa (dpaH)

H	-0.3244	0.5308	4.6265
C	0.0945	0.2360	5.6070
C	1.3868	0.4093	5.8183
C	-0.8984	-0.3242	6.5564
C	-0.5372	-0.7547	7.8553
C	-2.2501	-0.4342	6.1578
C	-1.5011	-1.2780	8.7257
H	0.5086	-0.6729	8.1720
C	-3.2152	-0.9590	7.0315
H	-2.5406	-0.1038	5.1538

C	-2.8451	-1.3830	8.3186
H	-1.2054	-1.6067	9.7278
H	-4.2578	-1.0365	6.7055
H	-3.5965	-1.7926	9.0014
C	2.7424	0.6254	5.9517
C	3.2532	1.8758	6.4644
C	3.7014	-0.3929	5.5907
C	4.6247	2.0778	6.5990
H	2.5427	2.6595	6.7433
C	5.0672	-0.1620	5.7363
H	3.3342	-1.3468	5.2005
C	5.5448	1.0683	6.2394
H	4.9903	3.0339	6.9886
H	5.7769	-0.9475	5.4551
H	6.6200	1.2382	6.3498

Stationary point:hydrosulfido cluster

Mo	0.5143	1.7224	0.3450
Mo	1.3694	-0.9746	0.4326
S	0.2043	0.0820	-1.3461
Cl	1.1997	-3.0373	-1.0358
Cl	2.5176	2.5124	-1.0048
Mo	-1.3350	-0.3385	0.4503
Cl	-3.0562	0.7537	-1.1240
N	-3.1841	-1.0222	1.4502
C	-3.6794	-2.2040	1.0401
C	-0.0493	4.7093	0.6325
N	0.7330	3.7620	1.1789
C	4.1700	-2.0519	1.0490
N	2.8945	-2.2233	1.4410
C	2.8951	-3.2307	2.3921
H	1.9778	-3.5745	2.8631
N	3.3148	-0.6382	-0.7088
H	3.3511	0.3530	-1.0152
H	3.2438	-1.2199	-1.5543
C	4.5471	-0.9979	0.0545
H	4.9065	-0.0919	0.5746
H	5.3548	-1.3325	-0.6179
N	4.9870	-2.9130	1.7161
C	6.4474	-2.9997	1.5975
H	6.7800	-3.9360	2.0637
H	6.7379	-3.0079	0.5367
H	6.9263	-2.1484	2.1053
C	4.1912	-3.6633	2.5746
H	4.6156	-4.4287	3.2192

N	-0.6893	3.0676	-1.0162
H	-1.5322	2.5405	-1.3159
H	-0.1192	3.2481	-1.8533
C	-1.0932	4.3593	-0.3816
H	-2.0678	4.2000	0.1143
H	-1.2288	5.1526	-1.1353
N	0.2333	5.9248	1.1783
C	-0.4289	7.1980	0.8702
H	-0.5079	7.3258	-0.2193
H	-1.4330	7.2298	1.3204
H	0.1797	8.0146	1.2796
C	1.5449	4.3907	2.1080
H	2.2878	3.8451	2.6838
C	1.2386	5.7353	2.1196
H	1.6419	6.5648	2.6945
N	-2.0242	-2.1792	-0.7182
H	-1.1813	-2.7018	-1.0200
H	-2.4993	-1.8330	-1.5621
C	-2.9394	-3.0645	0.0620
H	-3.6225	-3.6232	-0.5993
H	-2.3194	-3.8040	0.6001
N	-4.8563	-2.4647	1.6745
C	-5.6800	-3.6705	1.5293
H	-5.7698	-3.9360	0.4660
H	-5.2349	-4.5114	2.0833
H	-6.6808	-3.4596	1.9278
C	-5.1142	-1.4006	2.5311
C	-4.0730	-0.5094	2.3798
H	-3.9100	0.4528	2.8585
H	-6.0058	-1.3747	3.1520
S	-1.2826	1.5599	1.8161
S	-0.2358	-1.7723	1.9324
S	2.1364	0.8188	1.9575
H	1.3821	0.5707	3.0764

Hydride transfer

Stationary point: semihydrogenated dpa

H	-0.7851	0.5658	4.1735
C	-0.0464	0.4108	4.9893
C	1.2471	0.7482	4.7712
C	-0.7041	-0.1618	6.1933
C	-0.0097	-0.4785	7.3915
C	-2.1015	-0.4141	6.1717
C	-0.6795	-1.0147	8.5003
H	1.0683	-0.2948	7.4350

C	-2.7738	-0.9503	7.2801
H	-2.6619	-0.1787	5.2570
C	-2.0665	-1.2555	8.4566
H	-0.1153	-1.2485	9.4112
H	-3.8541	-1.1310	7.2270
H	-2.5859	-1.6740	9.3255
C	2.4173	0.6884	5.5736
C	2.8727	1.8119	6.3626
C	3.3331	-0.4299	5.5238
C	4.1056	1.8079	7.0212
H	2.2209	2.6915	6.4382
C	4.5617	-0.4159	6.1901
H	3.0435	-1.3126	4.9395
C	4.9787	0.6996	6.9503
H	4.3934	2.6891	7.6107
H	5.2116	-1.2989	6.1202
H	5.9460	0.7063	7.4632

Stationary point: hydrosulfido cluster

Mo	0.4841	1.7545	0.2917
Mo	1.3580	-1.0277	0.3511
S	0.2149	0.1061	-1.4002
Cl	1.3258	-2.9368	-1.1636
Cl	2.4917	2.5388	-0.9547
Mo	-1.3186	-0.3272	0.4108
Cl	-3.0360	0.7537	-1.0812
N	-3.1258	-1.0098	1.4599
C	-3.6333	-2.1962	1.0710
C	-0.0875	4.7350	0.5856
N	0.6782	3.7762	1.1379
C	4.1454	-2.0822	1.0668
N	2.8499	-2.2881	1.3760
C	2.8068	-3.3609	2.2532
H	1.8716	-3.7477	2.6490
N	3.3727	-0.5603	-0.6350
H	3.4235	0.4427	-0.8973
H	3.3600	-1.0930	-1.5158
C	4.5659	-0.9596	0.1728
H	4.8850	-0.0890	0.7726
H	5.4090	-1.2379	-0.4806
N	4.9296	-2.9824	1.7162
C	6.3963	-3.0540	1.6739
H	6.7082	-4.0259	2.0768
H	6.7450	-2.9706	0.6346
H	6.8364	-2.2471	2.2794

C	4.0952	-3.7951	2.4748
H	4.4897	-4.6007	3.0884
N	-0.6883	3.0939	-1.0782
H	-1.5327	2.5661	-1.3765
H	-0.1228	3.2630	-1.9213
C	-1.0979	4.3993	-0.4646
H	-2.0965	4.2629	-0.0128
H	-1.1845	5.1832	-1.2341
N	0.1807	5.9360	1.1633
C	-0.4724	7.2173	0.8639
H	-0.5281	7.3641	-0.2244
H	-1.4847	7.2411	1.2952
H	0.1305	8.0236	1.3008
C	1.4647	4.3830	2.1039
H	2.1903	3.8274	2.6918
C	1.1577	5.7264	2.1298
H	1.5445	6.5431	2.7333
N	-2.0384	-2.1665	-0.7430
H	-1.2344	-2.7080	-1.1006
H	-2.5462	-1.8093	-1.5637
C	-2.9355	-3.0548	0.0614
H	-3.6454	-3.5934	-0.5875
H	-2.3076	-3.8106	0.5655
N	-4.7819	-2.4565	1.7504
C	-5.6053	-3.6681	1.6468
H	-5.6627	-3.9887	0.5972
H	-5.1757	-4.4767	2.2577
H	-6.6168	-3.4348	2.0037
C	-5.0100	-1.3907	2.6132
C	-3.9801	-0.4955	2.4215
H	-3.8061	0.4675	2.8938
H	-5.8767	-1.3650	3.2682
S	-1.2615	1.5901	1.7650
S	-0.1942	-1.8008	1.8624
S	2.1832	0.8192	1.8353
H	1.4829	0.5519	2.9724
